# Oxygen-Vacancy-Engineered Pd-WO_3–*x*_ for Enhanced Plasma-Catalyzed CO_2_ Hydrogenation to CO

**DOI:** 10.1021/acscatal.5c07524

**Published:** 2026-01-14

**Authors:** Can Cheng, Yaolin Wang, Jia-nan Wang, Haomiao Xu, Wenjun Huang, Zhisong Liu, Wei Li, Yuyang Li, Zan Qu, Xin Tu, Naiqiang Yan

**Affiliations:** † School of Environmental Science and Engineering, 12474 Shanghai Jiao Tong University, Shanghai 200240, China; ‡ Department of Electrical Engineering and Electronics, School of Engineering, 4591 University of Liverpool, Liverpool L69 3GJ, U.K.; § School of Mechanical Engineering, 12474 Shanghai Jiao Tong University, Shanghai 200240, China; ∥ Shanghai Institute of Pollution Control and Ecological Security, Shanghai 200092, China

**Keywords:** plasma catalysis, CO_2_ hydrogenation, oxygen vacancies, Pd nanoparticles, reverse water−gas shift reaction

## Abstract

Plasma-catalytic CO_2_ hydrogenation to CO offers a promising route for carbon-neutral chemical synthesis. However, its advancement is constrained by low energy efficiency and limited mechanistic insight. Here, we developed an oxygen vacancy-engineered Pd-WO_3–*x*_ catalyst supported on nickel foam (NF), which exhibits enhanced performance in ambient plasma-driven CO_2_ conversion. The system leverages the conductive properties of NF to spatially divide the discharge zone into streamer and filamentary discharge zones, thereby enhancing plasma activation and interfacial charge transfer. Catalyst characterization reveals that Pd plays a critical role in stabilizing metastable oxygen vacancies (OVs) within WO_3–*x*_, which function as electron reservoirs to promote CO_2_ dissociation into CO. Density functional theory calculations and in situ spectroscopic studies confirm that Pd facilitates H_2_ dissociation, while vibrationally excited CO_2_ generated in the plasma gas phase preferentially adsorbs at OV sites. At a specific energy input of 33.6 kJ L^–1^, the system demonstrates superior performance, achieving 54.9% CO_2_ conversion with 99.8% selectivity toward CO, surpassing typical plasma-catalytic benchmarks. The catalyst exhibited good stability over 100 h of continuous operation, with a slight decrease in CO_2_ conversion (<10%) and nearly unchanged CO selectivity (>99%) due to strong metal–support interactions and the conductive nature of NF. This work demonstrates that OV engineering provides a promising strategy for designing efficient plasma-catalytic systems for CO_2_ conversion.

## Graphical abstract

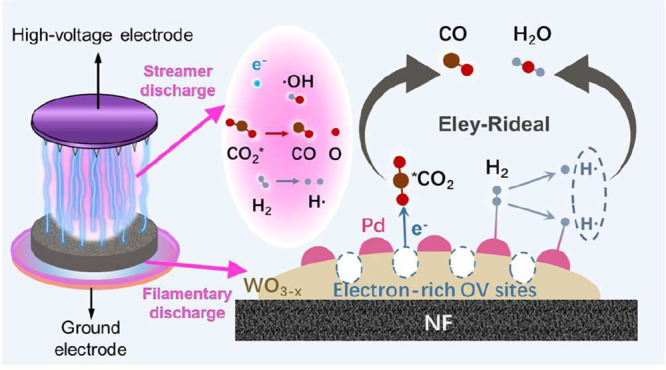

## 1 Introduction

Carbon dioxide (CO_2_) released from fossil fuel combustion and industrial processes is a major contributor to climate change. CO_2_ utilization offers a crucial technological pathway to achieve net zero.^1–4^ Among various CO_2_ utilization strategies, the reverse water–gas shift (RWGS) reaction is a key intermediate step in the synthesis of hydrocarbons and oxygenated compounds through downstream processes. However, conventional thermocatalytic RWGS requires high temperatures (>600 °C) to overcome the thermodynamic limitations of CO_2_ activation,^5–9^ leading to high energy consumption and catalyst deactivation due to sintering or coking.^2,10,11^ Although nonthermal plasma (NTP) catalysis has emerged as a promising approach to drive CO_2_ conversion under ambient conditions,^12–17^ existing systems tend to be less energy efficient, and the mechanism of plasma-catalyst synergy remains unclear.

Recent advances in plasma catalysis have demonstrated the potential of plasma catalysis to activate inert molecules such as CO_2_ and CH_4_ through electron-induced dissociation and vibrational excitation.^18–22^ However, most studies have focused on simple catalyst structures (e.g., bare metals or oxides) without active sites specifically designed for the plasma environment. For example, although oxygen vacancies (OVs) in reducible oxides (e.g., CeO_2_, TiO_2_) are known to enhance the adsorption and activation of thermally catalyzed CO_2_, their role in plasma-driven reactions is still not fully explored.^11,23–28^ This stems from the complexity of plasma-catalyst interactions, in which the electric field, active species, and transient surface states combine to control the reaction pathways. A recent study on nonmetallic plasma materials highlighted the key role of OVs in lowering the energy barrier for CO_2_ hydrogenation intermediates,^29^ yet their stability under plasma conditions remains a challenge. Similarly, studies of Cu/Al_2_O_3_ and Pt/Al_2_O_3_ catalysts in plasma-assisted CO_2_ hydrogenation have shown that altered surface oxygen species can significantly improve product selectivity,^30–32^ but energy efficiency remains suboptimal.

A key challenge lies in designing catalysts that synergistically lower the activation barrier with plasma-generated reactants while maintaining structural stability. Conventional approaches to improving the performance of RWGS, such as alloying noble metals (e.g., platinum, palladium) with reducible supports-tend to ignore the dynamic interactions between plasma-induced surface charges and defect-rich interfaces.^33–35^ For example, palladium-based catalysts have a strong CO_2_ adsorption capacity but tend to be more methanogenic than CO-selective under thermal conditions.^36–38^ In contrast, in a plasma environment, the presence of metastable OVs and electron-rich surfaces may redirect reaction pathways while inhibiting undesired side reactions. This hypothesis remains untested since systematic studies on OV engineering in plasma catalysis are limited, especially for RWGS. Recent breakthroughs in defect engineering, such as pressure-induced surface OV generation in WO_3_ ^39^ and supramolecular-enhanced localized electric fields in covalent organic frameworks (COFs),^40^ highlight the transformative potential for precise control of defect kinetics in catalytic systems. These advances suggest that combining OV-rich supports with plasma activation can lead to superior performance by modulating charge transfer and intermediate stabilization.

In this study, we demonstrate an effective RWGS reaction using plasma catalysis over Pd-WO_3–*x*_ supported on nickel foam (NF) with tunable oxygen vacancies, offering a promising strategy to address the efficiency bottleneck of this reaction under ambient conditions. Unlike conventional thermal catalytic systems, the WO_3–*x*_ support not only stabilizes Pd nanoparticles (NPs) but also serves as an electron reservoir, enabling dynamic charge compensation at the metal–support interface under plasma activation. Importantly, the spatial division of the discharge zone allows the catalyst to operate with a markedly reduced Pd loading, ensuring that the system remains economically feasible while maintaining high catalytic performance. The combination of oxygen-vacancy engineering and plasma activation thus provides a promising pathway for CO_2_ conversion, supporting the development of scalable carbon-utilization technologies aligned with global net-zero ambitions.

## 2 Experimental Section

### 2.1 Catalyst Preparation

Nickel foam substrates (diameter 30 mm, thickness 5 mm, pore size 130 ppi) were purchased from Guoxiang Environmental Materials Co., Ltd. They were immersed in 10% dilute hydrochloric acid for 20 min with ultrasonic pretreatment, rinsed with deionized water and dried. 0.43 g (NH_4_)_6_H_2_W_12_O_40_·6H_2_O (Macklin, 99.5%) was dissolved in 30 mL deionized (DI) water. The pH was adjusted to 1.2 with 3 M hydrochloric acid. The above solution along with the NF substrate was transferred to a 100 mL Teflon-lined stainless-steel autoclave and heated at 180 °C for 24 h. Subsequently, the product cooled to room temperature was cleaned several times using DI water and ethanol to remove residual compounds and dried overnight. The substrates were roasted in a muffle furnace at 500 °C for 1 h to obtain WO_3_/NF, which was continued to anneal in 5% H_2_/Ar at a rate of 5 °C min^–1^ and kept at 600 °C for 2 h. The products obtained were noted as WO_3–*x*_/NF. 50 mg Pd­(NH_3_)_4_Cl_2_·H_2_O (Aladdin, 98%) was dissolved in 30 mL DI water to form a pale yellow solution.WO_3_/NF was placed in the solution, subjected to vibration on an oscillating shaker, and dried overnight. Likewise, the obtained products were annealed at a rate of 5 °C min^–1^ and kept at 600 °C for 2 h to obtain Pd-WO_3–*x*_/NF. Figure 1a illustrates the preparation process of Pd-WO_3–*x*_/NF.

**Figure 1:**
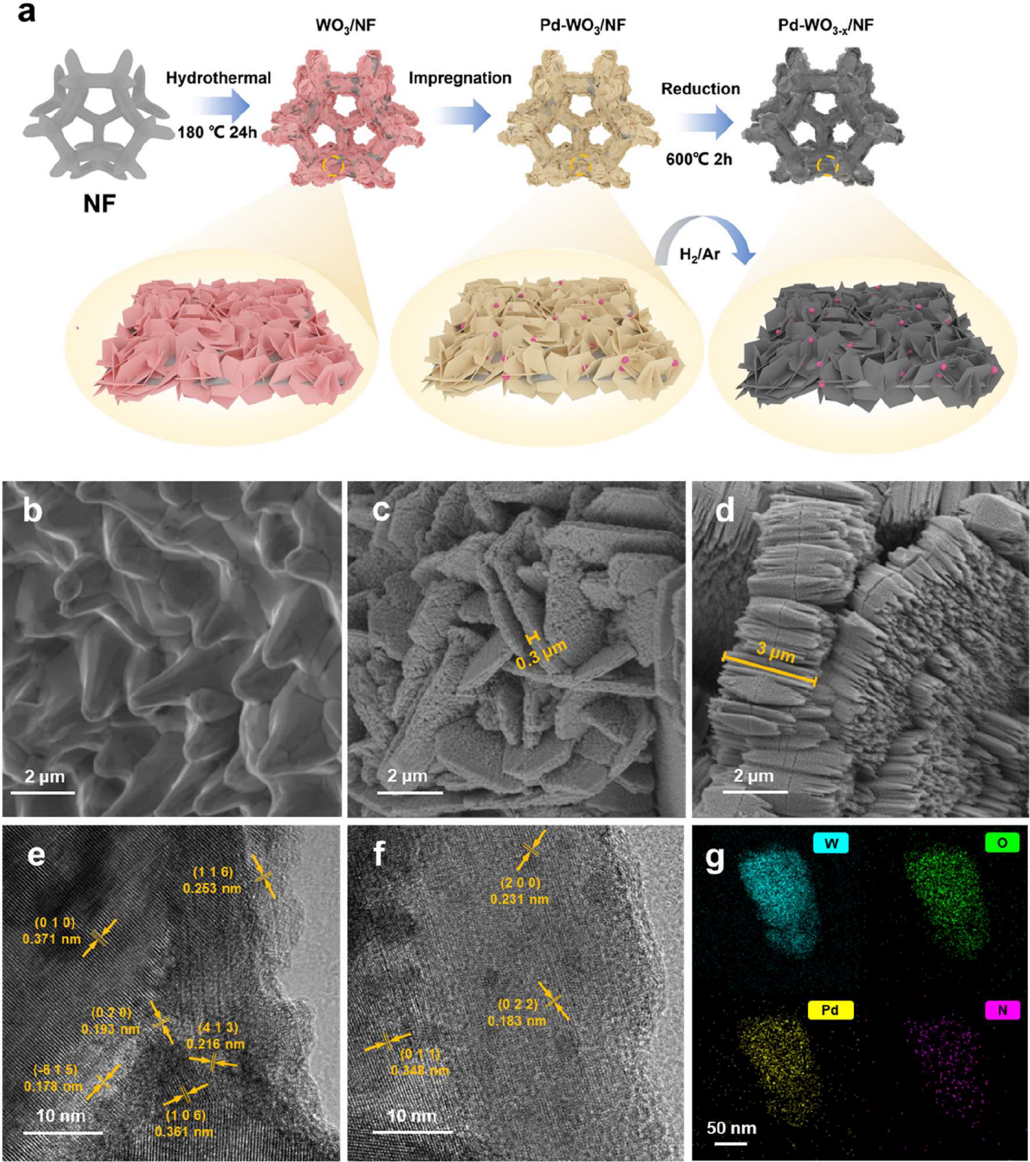
Synthesis and structural characterization of oxygen vacancy engineered Pd-WO_3–*x*_/NF catalysts. (a) Schematic illustration of the synthesis process for Pd-WO_3–*x*_/NF, (b–d) SEM images of (b) bare NF, (c) WO_3–*x*_/NF, (d) Pd-WO_3–*x*_/NF, (e, f) HRTEM images of WO_3–*x*_/NF and Pd-WO_3–*x*_/NF, (g) TEM-EDS mapping of Pd-WO_3*–x*_/NF.

### 2.2 Catalytic Evaluation

Plasma-catalytic CO_2_ hydrogenation experiments were conducted in a custom-designed cylindrical dielectric barrier discharge (DBD) reactor (30 mm i.d. × 34 mm o.d.), comprising three modular components: a tunable high-voltage electrode for dynamic adjustment of discharge geometry and gas flow distribution, a porous NF disk (28 mm diameter, 5 mm thickness) serving as both catalytic substrate and inductive material, and a grounded copper electrode (16 mm i.d. × 30 mm o.d.) positioned externally against the quartz barrier (Figure S1). The hollow high-voltage electrode (stainless steel 304; 29 mm o.d.) featured a conical tip with radial gas ports to ensure uniform gas dispersion across the streamer discharge zone (15 mm gap). The NF substrate, positioned adjacent to the quartz inner wall, defined the filamentary discharge zone, while the ground electrode was mechanically secured against the quartz barrier exterior by employing a polyether ether ketone gasket to ensure intimate electrical contact. Crucially, the axial displacement of the high-voltage electrode enabled precise tuning of the interelectrode gap (7–22 mm), optimizing plasma-catalyst coupling while maintaining parallel alignment between electrodes.

Figure S2 shows the schematic diagram of the experimental setup. The RWGS reaction was carried out at atmospheric pressure (101 kPa) without external heating, with a total gas flow rate of 50 mL min^–1^ (H_2_/CO_2_ = 3:2). The mass of Pd-WO_3–*x*_/NF and WO_3–*x*_/NF was ∼280 mg, corresponding to a weight hourly space velocity (WHSV) of 10,870 mL g^–1^ h^–1^. The Pd and W loadings (wt %) were determined by inductively coupled plasma optical emission spectroscopy (ICP-OES) (Table S1). Premixed gases were homogenized in a gas holder before entering the DBD reactor. Plasma was generated using a high-frequency alternating current (AC) high voltage power supply (Suman, CTP-2000KP) at 9.4 kHz, with specific energy input (SEI) varied from 14.4 to 33.6 kJ L^–1^. The voltage and current signals were sampled using a digital oscilloscope (Tektronix TBS 1000C). The gas products were quantified in real-time using a Fourier-transform infrared (FTIR) gas analyzer (MGA6 Plus, ABB), calibrated for CO_2_, CO and CH_4_. Optical emission spectroscopy (OES, Figspec FS-22) with a 25 μm slit and 0.5 s exposure resolved plasma-generated reactive species (400–900 nm), correlating discharge characteristics with catalytic performance (Figure S3).

### 2.3 Catalyst Characterization

Morphological features and elemental distributions were analyzed by scanning electron microscopy (SEM, TESCAN MIRA3) and high-resolution transmission electron microscopy (HRTEM, Thermo Fisher Talos F200X) operated at 200 kV. Elemental analysis was further performed by energy-dispersive X-ray spectroscopy (EDS) in both SEM and TEM to examine the dispersion of Pd, W, and O. N_2_ adsorption–desorption isotherms (Barrett–Joyner–Halenda method) and Brunaur-Emmett-Teller (BET) surfaces areas were acquired with a surface area analyzer (Quantachrome NOVA 2200E). Crystalline phases were determined by powder X-ray diffraction (PXRD, Shimadzu AS6100) using Cu–Kα radiation over a 2θ range of 10–80°, with lattice parameters refined via Rietveld analysis. Surface chemical states and OVs concentrations were quantified by X-ray photoelectron spectroscopy (XPS, Thermo Scientific K-Alpha) employing Al–Kα monochromatic radiation (hν = 1,486.6 eV), with adventitious carbon (C 1s at 284.8 eV) as an internal reference. Electron paramagnetic resonance (EPR, Bruker A300) spectroscopy was employed to characterize paramagnetic centers associated with OVs. Dielectric and conductivity properties critical for plasma-catalyst coupling were measured via broadband impedance spectroscopy (Agilent 4294A) across 6–12 kHz, with samples pretreated by silver paste coating and calcination to eliminate organic residues. Temperature-programmed desorption (TPD) and reduction (H_2_-TPR) experiments were performed on a Quantachrome AMI-90 and Micromeritics AutoChem II 2920, respectively. For CO_2_-TPD, prereduced catalysts were exposed to CO_2_ at 30 °C followed by He purging, and then heated to 900 °C at 10 °C min^–1^. H_2_-TPR profiles were acquired under 5% H_2_/Ar flow (30 mL min^–1^) with identical heating protocols. Raman vibrational modes were recorded on a Horiba LabRAM HR800 Evolution spectrometer using a 532 nm laser, resolving lattice distortions and defect states.

Plasma-catalytic surface intermediates were monitored using in situ Fourier-transform infrared spectroscopy (FTIR, Nicolet 6700) equipped with a custom-designed reaction cell. Prior to reaction, the catalysts were pretreated in an H_2_ plasma (10 W, 9.4 kHz) for 1 h. Subsequently, a CO_2_/H_2_/Ar gas mixture (10/15/20 mL min^–1^) was introduced into the cell. Transient experiments, involving alternating H_2_ and CO_2_ flows, were conducted to track the dynamic evolution of surface adsorbates, with spectra collected every 2 min (64 scans per spectrum). Detailed experimental protocols for in situ plasma-coupled FTIR measurement are provided in Figure S4.

### 2.4 DFT Calculations

Density functional theory (DFT) calculations were performed using the Vienna Ab Initio Simulation Package (VASP), employing the Perdew–Burke–Ernzerhof (PBE)-D3 functional and projector-augmented wave core potentials. Oxygen-deficient WO_3–*x*_ surfaces and Pd species anchored at vacancy sites, were constructed to reflect experimentally observed configurations. Adsorption energies, transition states, and charge redistribution were systematically investigated. Further computational details are provided in the Supporting Information.

## 3 Results and Discussion

### 3.1 Discharge-Favored Structure of Pd-WO_3–*x*_/NF

SEM reveals the morphological evolutions of bare NF, WO_3–*x*_ /NF and Pd-WO_3–*x*_/NF. Bare NF shows a smooth metallic framework (Figures 1b and S5), whereas WO_3–*x*_/NF displays a distinct lamellar morphology with an average thickness of about 0.3 μm, intergrown and superimposed on the NF skeleton (Figures 1c and S6). The introduction of Pd results in the coexistence of spindle-shaped structures with sharp ends (2–3 μm in length) and calcite-like polyhedra (10–20 μm in edge length) throughout the NF substrate (Figures 1d and S7). SEM-EDS analysis confirmed the uniform incorporation of Pd and W onto the NF substrate (Figure S8), consistent with the designed catalyst composition. The BET surface area of Pd-WO_3–*x*_/NF (14.3 m^2^ g^–1^) is higher than that of WO_3–*x*_/NF (11.8 m^2^ g^–1^) and bare NF (1.9 m^2^ g^–1^) (Figure S9). These results suggest that Pd modulates the crystallization behavior of WO_3–*x*_/NF and enhances the exposure of active sites, as further evidenced by the increase in CO chemisorption capacity from 75.2 (WO_3–*x*_/NF) to 213.5 μmol g^–1^ (Pd-WO_3–*x*_/NF) (Figure S10a and Table S2). The curvature radius of the spindle-shaped tip, being less than 50 nm, is predicted by theoretical studies to amplify the local electric field through geometric field enhancement at sharp protrusions.^41–43^ Electrical measurements reveal that Pd doping increased in the plasma peak current from 87.9 to 106.6 mA (Figures S11 and S12). Furthermore, OES reveals enhanced emission intensities of reactive species (e.g., *CO, H_α_) within the streamer discharge zone over Pd-WO_3–*x*_/NF (Figure S13), indicating that geometric field enhancement at the spindle tips promotes plasma activation.

The nanoscale structural effects induced by Pd were investigated using HRTEM. As shown in Figure 1e,f, both WO_3–*x*_/NF and Pd-WO_3–*x*_/NF display an ordered lattice structure with abundant surface steps and defects. WO_3–*x*_/NF exhibits well-defined lattice fringes with spacings of 0.371, 0.253, and 0.216 nm. In contrast, Pd-WO_3–*x*_/NF shows compressed lattice spacings (0.348 nm, 0.231 nm, 0.183 nm) and pronounced lattice distortions (Figure 1f), indicating electron transfer from Pd to W atoms.^44,45^ TEM-EDS mapping (Figure 1g) reveals highly dispersed Pd NPs, while tungsten (W) and oxygen (O) remain uniformly distributed on the support, suggesting strong interfacial contact between Pd NPs and the WO_3–*x*_ support that likely facilitates efficient electron transport under plasma activation. HRTEM analysis (Figure S14a) further confirms that Pd NPs have a narrow size distribution with an average diameter of ∼2.3 ± 0.08 nm, demonstrating uniform dispersion without agglomeration on the WO_3–*x*_/NF carrier. Notably, the absence of Pd sintering indicates that the oxygen-deficient WO_3–*x*_ support effectively stabilizes the Pd active sites, which is crucial for maintaining catalytic stability during plasma reactions. This stabilization effect is further supported by CO-TPD measurements and postreaction microstructural analysis (Figures S10b, S14b and Table S2). The hierarchical structure, featuring spindle-like protrusions and a robust metal–support interface, integrates multiple advantageous features that collectively enhance plasma-driven catalytic activity and durability.

The XRD analysis reveals a clear evolution of crystal phases during the reduction process. Pristine WO_3_ (monoclinic, JCPDS#97-003-1823) is predominantly converted to oxygen-deficient WO_2.9_ (JCPDS#97-002-4736) upon thermal reduction, resulting in the formation of partial OVs (Figure S15). The incorporation of Pd induces a further phase transition to monoclinic WO_2_ (JCPDS #97-000-8217). As a result of this reduction, the unit cell volume of Pd-WO_3–*x*_/NF contracts by 6.7%, and the average crystalline grain size decreases from 32.1 nm in WO_3–*x*_/NF to 14.6 nm. These results indicate that the introduction of Pd NPs disrupts the long-range crystalline order of WO_3–*x*_/NF and promotes the formation of shear planes and local lattice distortion. This structural rearrangement is believed to be critical for the stabilization of OVs during plasma-catalytic RWGS reaction, thereby enhancing catalyst stability and performance under reactive plasma conditions.

### 3.2 Oxygen Vacancy-Tuned Electronic States

XPS and EPR analyses are employed to investigate the electronic states of the catalysts. The W 4f spectrum of pristine WO_3_ (Figure 2a) shows the characteristic W^6+^ doublet at 35.3 (4f_7/2_) and 37.4 eV (4f_5/2_), with no detectable low-valence W peaks. After thermal reduction, the W^6+^ peaks shift significantly toward higher binding energies, appearing at 36.2 eV (4f_7/2_) and 37.8 eV (4f_5/2_). This shift arises from the altered coordination environment of W species caused by O loss, which decreases the local electron density and induces a chemical shift in binding energies. Moreover, additional peaks corresponding to mixed-valence states W^5+^ (34.6 eV, 4f_5/2_) and W^4+^ (32.5 eV, 4f_5/2_) appear, indicating the formation of OV. The introduction of Pd NPs further enhances the relative intensity of low-valent W species (W^4+^ and W^5+^). This effect is attributed to to electron donation from Pd NPs to WO_3–*x*_, which stabilizes low-valent W species and suppresses the reoxidation of W^4+^ to W^6+^. Furthermore, the O_def_/O_lat_ ratio increases to 0.42 (Figure 2b), confirming the enhanced OV concentration upon Pd incorporation. The evolution of defect states is further supported by EPR spectroscopy (Figure 2c). All reduced samples exhibit a strong signal at *g* = 2.003, attributed to unpaired electrons localized on W^5+^ sites adjacent to OVs – indicative of paramagnetic centers formed by electron trapping. Notably, Pd introduction increases the EPR signal intensity by 38%, and shifts the resonance field from 3502.5 to 3503.4 G, indicating stronger spin–orbit coupling at the OV sites as a result of Pd-mediated electron transfer. By comparing the Raman spectra of WO_3_/NF, WO_3–*x*_/NF and Pd-WO_3–*x*_/NF (Figure 2d), the stretching vibrational peak of the W–O–W bond is red-shifted from 810 (WO_3_) to 805 cm^–1^ (WO_3–*x*_), and the bending vibrational peak of the O–W–O bond shifts from 276 to 262 cm^–1^. These spectral shifts indicate lattice strain relaxation induced by electron transfer from Pd NPs to the WO_3–*x*_ framework. Moreover, the appearance of the edge-sharing WO_6_ mode at 705 cm^–1^ suggests the formation of a structurally robust framework that promotes plasma-catalytic CO_2_ hydrogenation.^46^

**Figure 2:**
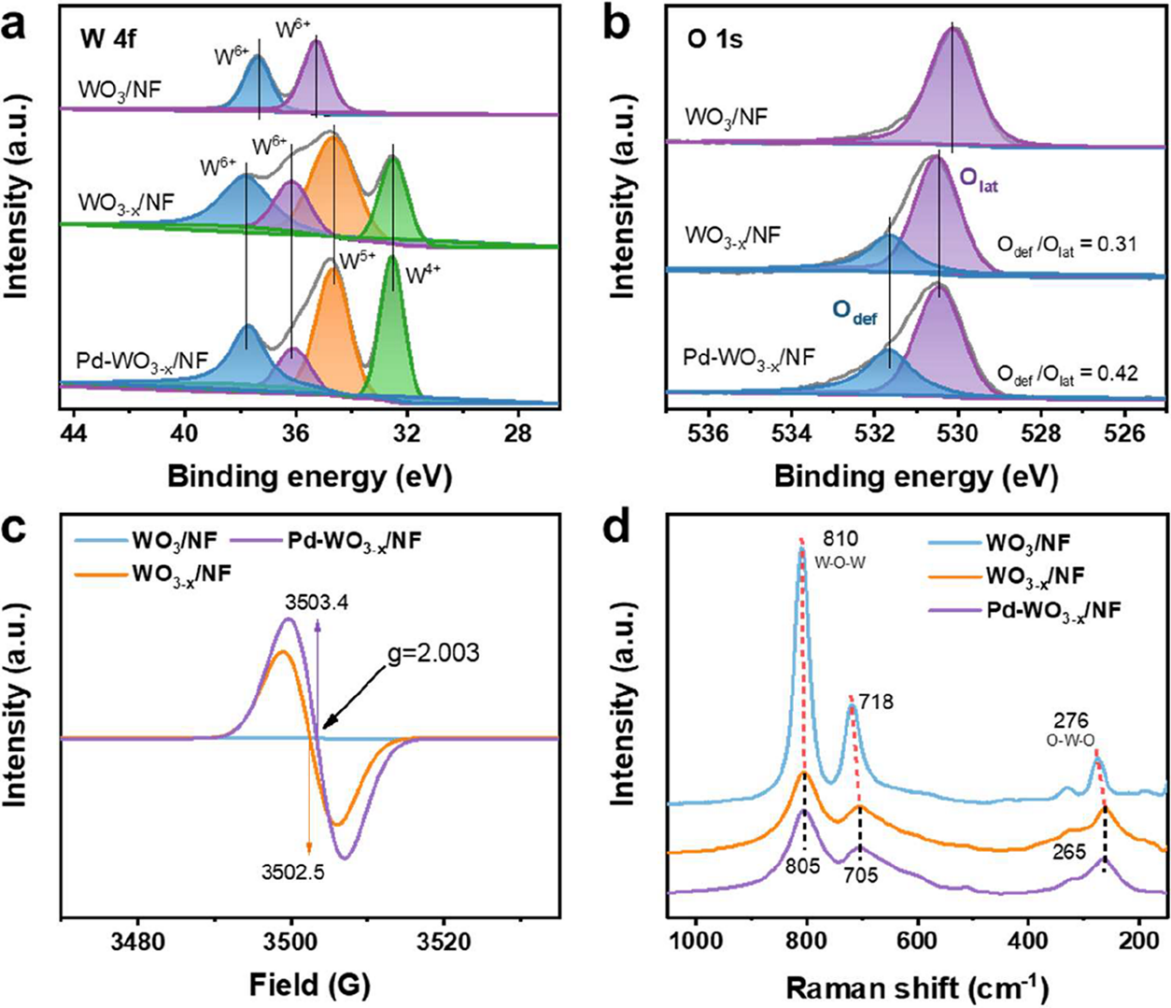
Oxygen vacancy engineering and electronic state modulation in Pd-WO_3–*x*_/NF. (a–c) XPS and EPR analyses of WO_3_/NF, WO_3–*x*_/NF vs Pd-WO_3–*x*_/NF: (a) XPS spectra of W 4f and (b) O 1s, (c) EPR spectra. (d) Raman spectra of WO_3_/NF, WO_3–*x*_/NF vs Pd-WO_3–*x*_/NF.

H_2_-TPR expertiments (Figure S16) reveal the evolution of W valence states upon Pd loading. The presence of OVs in WO_3–*x*_ lowers the primary W reduction temperature by 89 °C. Pd introduces distinct reduction peaks at 353 °C (PdO reduction) and 577 °C (W^6+^ transitioning to W^4+^). The total H_2_ consumption decreases by 22% over WO_3–*x*_/NF, which can be attributed to the increased concentration of OVs (Table S3). These results suggest a strong interaction between Pd and the WO_3–*x*_ support, which stabilizes reduced W oxidation states. CO_2_ temperature-programmed desorption (CO_2_-TPD, Figure S17) indicates a unique synergistic effect between OVs and Pd on the redistribution of surface basic sites. New medium-strength basic sites (444**–**559 °C) emerge, while the high temperature site characteristic of WO_3–*x*_/NF is retained. Notably, the loading of Pd NPs results in the appearance of a major interfacial basic center at 412 °C, reflecting a new alkaline interaction at the Pd-support interface. The increased total surface alkalinity (Table S4) further supports Pd-mediated optimization of surface basic site distribution.

Under plasma conditions, electron transfer from Pd NPs to adjacent W atoms induces compressive lattice strain in WO_3–*x*_, stabilizing metastable OVs. The shear-state WO_2_ phase, characterized by edge-sharing WO_6_ octahedra, confines OVs within its distorted lattice framework, while Pd NPs act as both H_2_ dissociation sites and electron donors. This synergistic design promotes charge localization (e.g., spindle-tip feature), defect stabilization via lattice strain and interfacial charge compensation, collectively addressing the challenges of OV stability and the formation of plasma-compatible active sites.

### 3.3 Plasma-Catalytic CO_2_ Hydrogenation

To elucidate the critical role of OVs in Pd-WO_3–*x*_/NF during plasma-catalytic RWGS reactions, systematic catalytic evaluations are conducted at an SEI of 33.6 kJ L^–1^ (Figure S18). In a reactor with a 15 mm streamer discharge zone (Figure S1), the NF-supported system achieves a CO_2_ conversion of 37.8%–double that of systems using alternative conductive supports such as alumina foam (AF) and silicon carbide foam (SF) (Figures 3a and S19). This enhanced performance is attributed entirely to the electric properties of NF, which sustain high-intensity streamer discharge through optimized electron avalanche propagation and the generation of radical species.^47^ Catalytic performance is assessed at a WHSV of 10,870 mL g^–1^ h^–1^ (Figure 3b). The CO_2_ conversion of WO_3–*x*_/NF reaches 41.8% (a 10.6% conversion gain over bare NF), Pd-WO_3–*x*_/NF achieves outstanding performance, with CO_2_ conversion plateauing at 54.9%, approximately 9 times higher than that using plasma-only. High CO selectivity (99.8%) and a CO production rate of 440.2 mmol g^–1^ h^–1^ confirm the suppression of methanation and other side reactions. Even in the absence of streamer discharge, Pd-WO_3–*x*_/NF achieved a 66% conversion gain over bare NF (Figure S20), demonstrating a significant intrinsic catalytic contribution.

**Figure 3:**
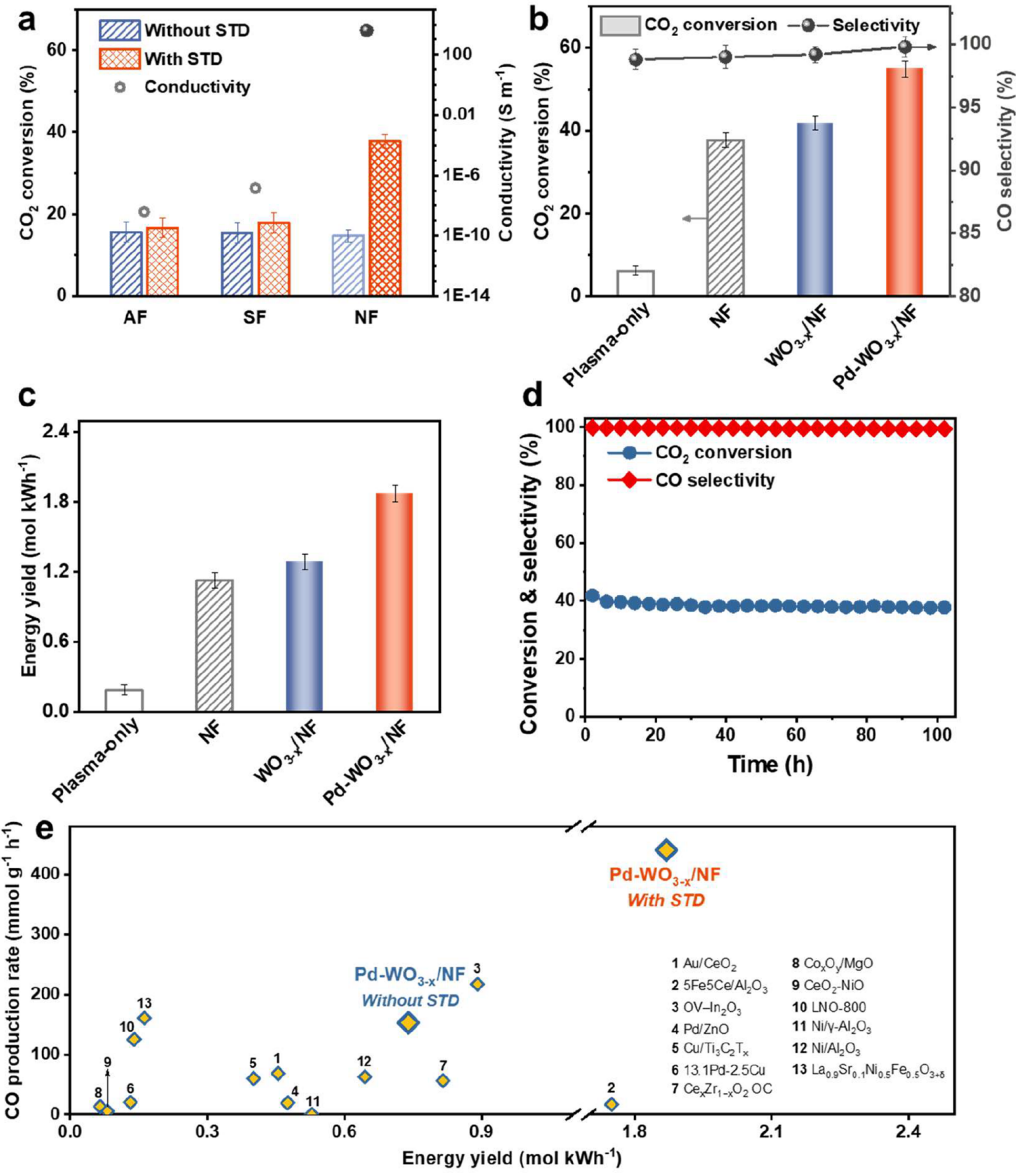
Performance of plasma-catalytic CO_2_ hydrogenation. (a) CO_2_ conversion without and with the streamer discharge zone for different conductivity substrates (AF, SF, and NF), (b) comparison of CO_2_ conversion and CO selectivity for plasma-only, and plasma coupled with NF, WO_3–*x*_/NF, and Pd-WO_3–*x*_/NF, (c) comparison of energy yield for plasma-only, and plasma coupled with NF, WO_3–*x*_/NF, and Pd-WO_3–*x*_/NF, (d) stability of CO_2_ conversion and CO selectivity over Pd-WO_3–*x*_/NF, (e) reported energy yield vs CO production rate over different catalysts for plasma-catalytic CO_2_ hydrogenation. Pd-WO_3–*x*_/NF with and without the streamer discharge (STD) are compared. Detailed reaction conditions are summarized in Table S5.

Energy yield measurements (Figure 3c) further confirm the superior performance of the Pd-WO_3–*x*_/NF catalyst. At 14.4 kJ L^–1^, the energy yield reaches 1.87 mol kWh^–1^, representing a 65% improvement over the plasma-only condition, and a 45% enhancement compared to the WO_3–*x*_/NF catalyst. This enhancement can be attributed to the synergistic effects of improved streamer discharge, driven by the apparent dielectric response of the NF, and enhanced surface reaction kinetics facilitated by OV-mediated catalytic pathways. The incorporation of NF significantly increases the amplitude of the pulse current from 39.7 to 99.4 mA (Figure S11), while the peak voltage decreases from 17.4 to 13.8 kV (Figure S12), indicating improved charge transfer efficiency compared to the plasma-only condition.^48^ In addition, a WHSV of 10,870 mL g^–1^ h^–1^, a H_2_/CO_2_ ratio of 3:2 and a frequency of 9.4 kHz are the preferred conditions for Pd-WO_3–*x*_/NF to achieve excellent performance (Figures S21–S24).

The stability of the Pd-WO_3–*x*_/NF catalyst in the plasma-catalytic RWGS reaction was carefully evaluated. As shown in Figure 3d, the catalyst maintains robust performance over 100 h of continuous operation, with only a slight decrease in CO_2_ conversion (<10%) and nearly constant CO selectivity (99.3%). Postreaction characterization confirms partial oxidation of OVs but preservation of W^5+^/W^4+^ states and structural integrity (Figures S25–S31), which is attributed to the robust metal–support interaction between Pd and WO_3–*x*._ ^49,50^ Comparative analysis with recently reported catalysts (Figure 3e, Table S5) shows that Pd-WO_3–*x*_/NF outperforms other plasma-catalytic systems in CO_2_ conversion and energy yield. Notably, the plasma-catalytic performance of Pd-WO_3–*x*_/NF significantly outperforms its thermal activation counterpart at temperatures above 400 °C, even without enhancements in streamer discharge (Figure S32). The integrated plasma-catalyst system offers exceptional compatibility and flexibility with intermittent renewable energy sources, enabling rapid response to power adjustments while maintaining stable performance, which is critical for enhancing the stability and flexibility of large-scale power grids.

### 3.4 Mechanistic Insights

We systematically investigate the dynamic interaction among OVs, Pd-WO_3–*x*_ interfaces, and plasma-generated reactive species using in situ plasma-coupled FTIR spectroscopy to identify surface intermediates during plasma-catalytic CO_2_ hydrogenation (Figure S4). Upon introducing CO_2_, we observe vibrational bands corresponding to gaseous CO at 2169 and 2119 cm^–1^,^51,28^ linearly chemisorbed CO at 2077 cm^–1^,^11,52^ and H_2_O-related bands at 3560–3680 cm^–1^ (asymmetric/symmetric O–H stretching)^53,54^ and 1635 cm^–1^ (bending vibration).^55,56^ Additionally, CO_2_ adsorption induces changes in the surface hydroxyl environment of the catalyst, resulting in the appearance of stretching vibration peaks for surface free hydroxyl (−OH) groups in the 3728–3600 cm^–1^ range. This phenomenon correlates with the redistribution of hydroxyl groups caused by CO_2_ adsorption. Simultaneously, the asymmetric stretching vibration peaks of gaseous CO_2_ are distinctly observed at 2360 and 2340 cm^–1^ (Figure 4a,b, red line).^48,57^ No characteristic peaks for chemisorbed CO_2_ species (e.g., carbonates, bicarbonates) within the 1300–1600 cm^–1^ range are detected, further supporting the direct dissociation pathway of CO_2_. Time-resolved analysis reveals that CO bands on both WO_3–*x*_/NF and Pd-WO_3–*x*_/NF reach saturation within 3 min, indicating rapid CO_2_ dissociation. Notably, Pd-WO_3–*x*_/NF exhibits 2.3 times higher integrated CO band intensities than WO_3–*x*_/NF (Figure 4a,b), directly correlating with its superior catalytic activity. Subsequent H_2_ introduction leads to a simultaneous decrease in both CO and CO_2_ signals, while H_2_O signals accumulate (Figure 4a,b, from deep blue to shallow blue lines). The absence of formate (2898, 1594, 1393 cm^–1^) or carboxylate intermediates excludes formate-mediated pathways, supporting a direct surface CO_2_ dissociation mechanism.^58^ Control experiments with reversed gas sequencing (H_2_ pretreatment followed by CO_2_) result in negligible CO production (Figure S33), suggesting that plasma-activated CO_2_ is the primary adsorbed species and reacts with H atoms via an Eley–Rideal mechanisms.

**Figure 4:**
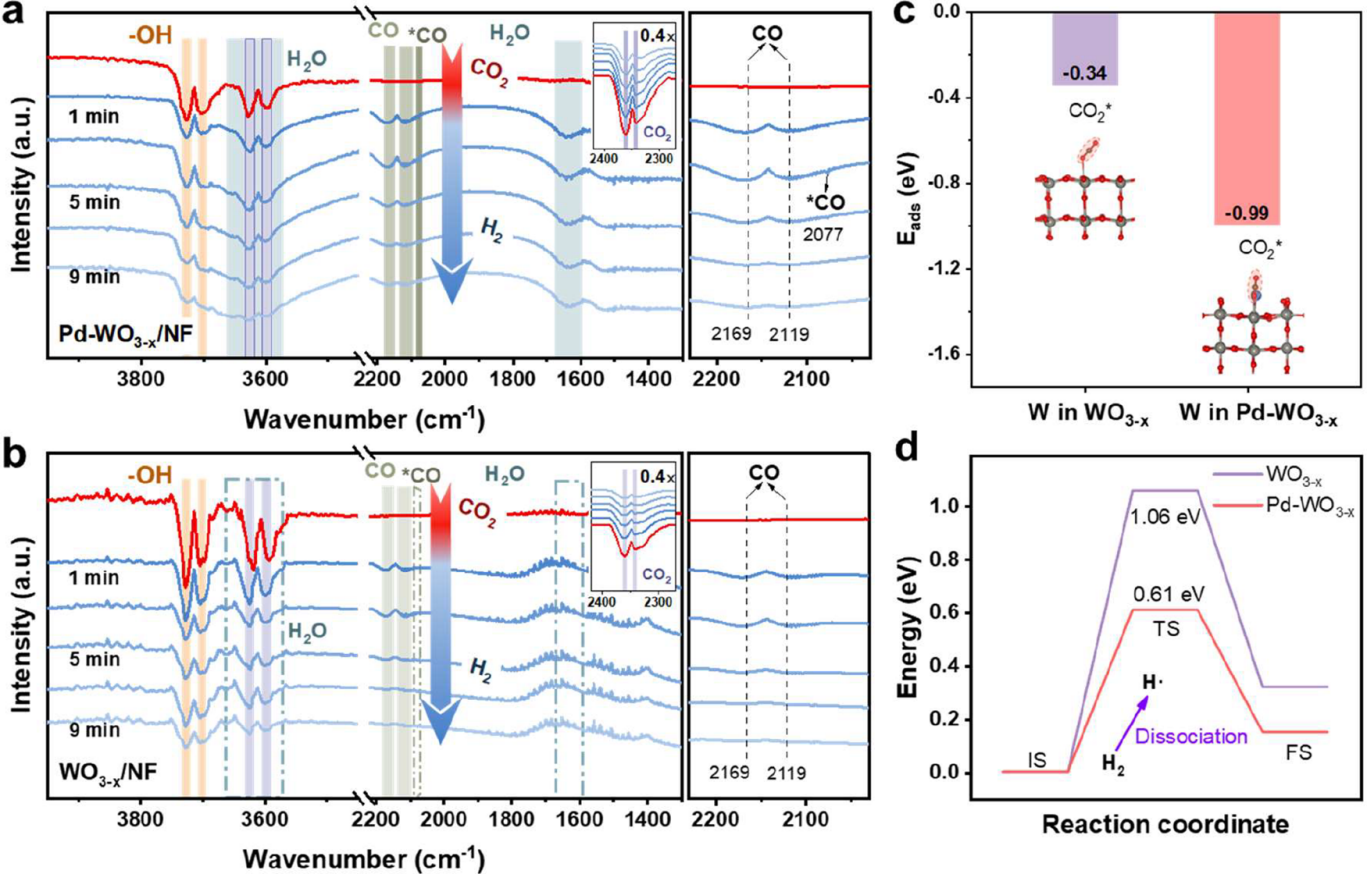
In situ FTIR characterization and DFT calculation results of plasma-catalytic CO_2_ hydrogenation. In situ FTIR spectra of surface species on (a, b) WO_3–*x*_/NF and Pd-WO_3–*x*_/NF where the CO_2_ and H_2_ mixture was introduced at *Q*_gas_ = 30 mL min^–1^. Recording interval = 2 min, (c) comparison of CO_2_ adsorption energies on WO_3–*x*_ and Pd-WO_3–*x*_/NF, (d) dissociation energy pathways of H_2_ on WO_3–*x*_/NF and Pd-WO_3–*x*_/NF.

To further elucidate the role of Pd-WO_3–*x*_/NF interface in mediating reactive species during CO_2_ hydrogenation, we employ OES to monitor the distribution of key species (e.g., CO, H_α_) in the filamentary discharge zone (Figure S3 and Table S6). Unlike the global plasma excitation observed in the streamer discharge zone, the discharge intensity near the catalyst surface is significantly reduced, revealing the localized modulation of active species by Pd-WO_3–*x*_/NF (Figure S34). In addition, reduced H_α_ emissions in Pd-WO_3–*x*_/NF (Figure S35) alongside increased CO-related emissions, suggest that surface-catalyzed reactions consume H, consistent with the in situ FTIR results. The suppression of carbon-related emissions at 612.0 nm further confirms the ability of the catalyst to mitigate carbon deposition. The coupling of H_2_ dissociation facilitated by Pd and *CO_2_ adsorption stabilized by OVs inhibits competing surface recombination pathways at the catalyst surface, leading to selective CO formation while inhibiting undesired carbon-forming side reactions. This reactor design couples streamer discharge with catalyst stability, optimizing plasma energy utilization.

DFT calculations corroborate the in situ plasma-coupled FTIR and OES observations. Structural optimization reveals geometric differences between WO_3–*x*_ and Pd-WO_3–*x*_ (Figure S36). Higher CO_2_ adsorption energy on Pd-WO_3–*x*_ (vs WO_3–*x*_) confirms that the FTIR-detected CO_2_* stabilization at OVs (Figure 4c). Weaker H_2_ adsorption enables preferential adsorption of vibrationally excited CO_2_ (Figure S37 and Table S7), consistent with the Eley–Rideal mechanism. Furthermore, enhanced CO adsorption energy on Pd-WO_3–*x*_ helps suppress overhydrogenation (Figure S38 and Table S7). The calculated H_2_ dissociation barrier supports this mechanism (Figures 4d, S39 and S40). On Pd-WO_3–*x*_, the activation energy of H_2_ dissociation is significantly lower than that on WO_3–*x*_, indicating that Pd NPs efficiently dissociate H_2_ into H atoms. This synergistic effect is attributed to the strong metal-support interaction (SMSI) between Pd and WO_3–*x*_, which polarize the Pd surface and facilitates electron transfer to adsorbed H_2_ molecules. The reduced H_2_ dissociation barrier on Pd-WO_3–*x*_ indicates enhanced hydrogen spillover and suppressed over-reduction. In summary, Pd NPs promote both CO_2_ and CO adsorption and H_2_ dissociation, driving the overall reaction pathway and accounting for the superior plasma-catalytic performance of Pd-WO_3–*x*_/NF.

DFT calculations demonstrate the interfacial synergy between Pd and WO_3–*x*_ for CO_2_ activation.^59–61^ Charge density difference analysis shows that both WO_3–*x*_ and Pd-WO_3–*x*_ donate electrons to CO_2_, with charge transfer at the interface increasing from 0.023 e^–^ (WO_3–*x*_) to 0.043 e^–^ (Pd-WO_3–*x*_) (Figure 5a). This enhanced polarization is associated with stronger CO_2_ adsorption and W–O bonding. Crystal orbital Hamiltonian population (COHP) analysis confirms the enhancement of covalent interactions, with W–O bond strength increasing from 0.42 eV for WO_3–*x*_ to 0.70 eV for Pd-WO_3–*x*_ (Figure 5b). Simultaneously, the W d band center shifts from −0.096 to 0.080 eV (Figure 5c), promoting greater electron backdonation into the CO_2_ 2π* orbitals. These changes at the Pd-WO_3–*x*_ interface lower the CO_2_ dissociation barriers while maintaining structural stability.

**Figure 5:**
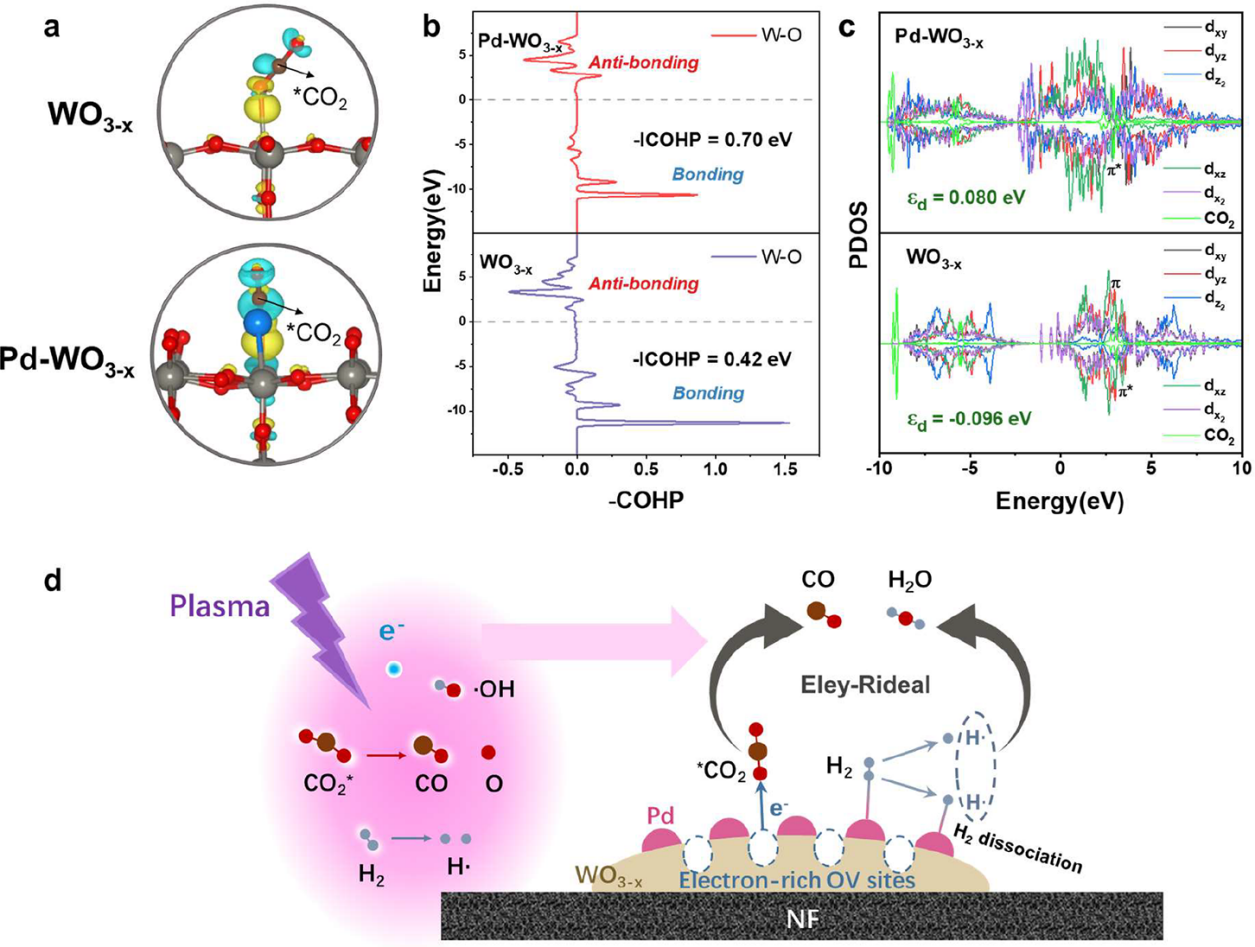
DFT calculations of catalysts for plasma-catalytic CO_2_ hydrogenation. (a) Charge density difference on WO_3–*x*_ and Pd-WO_3–*x*_ models, cyan and yellow regions represent the electron accumulation and depletion, respectively, gray, blue, red and brown balls represent W, Pd, O, and C atoms, respectively, (b) crystal orbital Hamilton population analysis of W–O bonds at the interface for WO_3–*x*_ and Pd-WO_3–*x*_, (c) PDOS spectra and corresponding d-band centers of WO_3–*x*_ and Pd-WO_3–*x*_ models. (d) Synergistic mechanism of oxygen-vacancy-engineered Pd-WO_3–*x*_ for plasma-catalytic CO_2_ hydrogenation.

Projected density of states (PDOS) analysis provides further insights into the π-backdonation mechanisms driving CO_2_ activation. For WO_3–*x*_, the C=O π* antibonding orbitals of CO_2_ interact side-on with W 5d_*yz*_/d_*xz*_ orbitals, forming localized electronic states near the Fermi level (−1.5–0 eV, Figure 5c). These π-backdonation interactions lead to modest charge transfer and weak adsorptions. The introduction of Pd induces delocalized hybrid bands between −2.8 and −0.5 eV, primarily resulting from the redistribution of W 5d_*xz*_ orbitals, rather than direct d–CO_2_ orbitals from Pd. Further calculations (in the Supporting Information) confirm negligible hybridization between Pd 4d states and CO_2_ π* orbitals (Figure S41). Instead of directly participating, Pd atoms modulate the electronic structure of adjacent W sites via SMSI, elevating the energy of W d_*xz*_ orbitals. This electronic rearrangement enhances π-backdonation from W d_*xz*_ orbitals to the CO_2_ π* orbitals. The resultant metallic-like conductivity arises from Pd-mediated delocalization of W d_*xz*_ orbitals, corroborated by Bader charge analysis showing 0.02 e^–^ accumulation at W sites. These interfacial electron reservoirs facilitate rapid charge transfer from plasma-generated hot electrons to CO_2_ adsorbates, supporting the high activity observed experimentally.

In summary, plasma-generated electrons are injected into OVs on WO_3–*x*_, while vibrationally excited CO_2_ molecules preferentially adsorb at these defect sites. The presence of OVs induces asymmetric charge redistribution, polarizing the C–O bonds and lowering their dissociation energy. Concurrently, Pd NPs dissociate H_2_ into H atoms, which spill over onto WO_3–*x*_ through the Pd–W interface, leading to CO formation and facilitating H_2_O desorption (Figure 5d). The spatial decoupling of plasma excitation ensures that the energetic electron avalanche is confined to the streamer discharge zone, while the catalyst surface experiences a milder discharge intensity. This configuration enhances plasma activation, minimizes plasma-induced catalyst degradation, and enables stable, long-term operation with high energy yield.

## 4 Conclusions

This study demonstrates the effectiveness of oxygen-vacancy-engineered Pd-WO_3–*x*_/NF catalysts for plasma-assisted CO_2_ hydrogenation under ambient conditions, achieving both high efficiency and long-term stability. The incorporation of Pd NPs onto oxygen-deficient WO_3–*x*_ creates a dynamic catalytic interface, where plasma-generated reactive species synergistically activate both CO_2_ and H_2_. Comprehensive structural and electronic characterizations reveal that Pd stabilizes metastable oxygen vacancies, induces lattice strain, and triggers electronic reconstruction. These effects enhance CO_2_ adsorption, weaken C–O bonds, and reduce the likelihood of over-reduction. Experimental observations, supported by DFT calculations, indicate that vibrationally excited CO_2_ generated by plasma interacts synergistically with OV sites, facilitating direct dissociation into *CO intermediates and suppressing deeper hydrogenation pathways such as formate and carboxylate formation. Concurrently, Pd NPs effectively dissociate H_2_, enabling selective hydrogenation and promoting CO formation. The reactor design strategically exploits the electric field properties of the NTP to sustain streamer discharge while spatially decoupling plasma excitation zones from the catalytic surface. This spatial separation minimizes catalyst degradation, thereby preserving catalytic performance and stability. The system exhibits an energy yield of 1.87 mol kWh^–1^, outperforming conventional packed-bed plasma-catalytic reactors. The observed catalyst stability is attributed to SMSI stabilized by OVs, which enable electronic delocalization across the Pd-WO_3–*x*_ interface while maintaining active sites. The interfacial charge redistribution and d-band center modulation described herein offer a broadly applicable strategy for the rational design of plasma-catalytic systems. By integrating defect engineering with plasma catalysis, this work presents a promising strategy for energy-efficient CO_2_ conversion, contributing to the development of scalable carbon utilization technologies aligned with global net zero targets.

## Supplementary Material



## Notes

Supplementary experimental section, supplementary catalyst characterization (ICP-OES, SEM, BET, and CO-TPD), plasma electrical and optical diagnostics, supplementary catalyst characterization (HRTEM, XRD, H_2_-TPR, and CO_2_-TPD), supplementary plasma-catalytic performance, supplementary characterization of spent Pd-WO_3–*x*_/NF catalysts, supplementary in situ characterization and diagnostic results, and details of DFT calculations (DOCX)
