# Supplementary material for: Oxygen-Vacancy-Engineered Pd-WO3–x for Enhanced Plasma-Catalyzed CO2 Hydrogenation to CO
Source: ACS Catal. 2026 Jan 14;16(3):2395–405. doi: 10.1021/acscatal.5c07524 (PMC12888645; doi:10.1021/acscatal.5c07524)
Supplement: Supplementary file 1 [file cs5c07524_si_001.docx]

**Supporting Information**

**Oxygen-Vacancy-Engineered Pd-WO_3-x_ for Enhanced Plasma-Catalyzed CO_2_ Hydrogenation to CO**

Can Cheng^a, §^, Yaolin Wang^b, §^, Jia-nan Wang^a*^, Haomiao Xu^a^, Wenjun Huang^a^, Zhisong Liu^a^, Wei Li^c^, Yuyang Li^c^, Zan Qu^a, d^, Xin Tu^b*^ and Naiqiang Yan^a, d*^

^a^ School of Environmental Science and Engineering, Shanghai Jiao Tong University, Shanghai 200240, China

^b^ Department of Electrical Engineering and Electronics, School of Engineering, University of Liverpool, Liverpool L69 3GJ, UK

^c^ School of Mechanical Engineering, Shanghai Jiao Tong University, Shanghai 200240, China

^d^ Shanghai Institute of Pollution Control and Ecological Security, Shanghai 200092, China

^*^Corresponding authors

wangjianan9591@sjtu.edu.cn (Jia-nan Wang)

xin.tu@liverpool.ac.uk (Xin Tu).

nqyan@sjtu.edu.cn (Naiqiang Yan).

^§^ These authors contributed equally to this work

**Table of contents**

**S1 Supplementary experimental section**

S1.1 Chemicals 1

S1.2 Definition of key performance metrics 1

S1.3 Experimental setup 2

S1.4 Computational details 5

**S2 Supplementary catalyst characterization (ICP-OES, SEM, BET and CO-TPD)**

S2.1 ICP-OES analysis of the catalysts 7

S2.2 SEM analysis of catalyst morphology 7

S2.3 BET analysis of the catalysts 9

S2.4 CO-TPD analysis 9

**S3 Plasma electrical and optical diagnostics**

S3.1 Electrical signal analysis 11

S3.2 Optical emission spectroscopic diagnostics 13

**S4 Supplementary catalyst characterization (HRTEM, XRD, H_2_-TPR and CO_2_-TPD)**

S4.1 HRTEM analysis of the catalysts 14

S4.2 XRD analysis 14

S4.3 H_2_-TPR analysis 15

S4.4 CO_2_-TPD analysis 15

**S5 Supplementary plasma-catalytic performance**

S5.1 CO_2_ conversion using different catalysts and substrates 17

S5.2 Effect of discharge gap on CO_2_ conversion 18

S5.3 Effect of WHSV on CO_2_ conversion 19

S5.4 Effect of H_2_/CO_2_ ratio and discharge frequency on plasma-catalytic CO_2_ hydrogenation

20

**S6 Supplementary characterization of spent Pd-WO_3-x_/NF catalysts**

S6.1 XPS analysis of fresh and spent catalysts 22

S6.2 O_2_-TPO analysis of fresh and spent catalysts 23

S6.3 SEM and HRTEM characterization of fresh and spent catalysts 24

S6.4 XRD analysis of fresh and spent catalysts 25

S6.5 EPR analysis of fresh and spent catalysts 25

S6.6 O_2_-TPD analysis of fresh and spent catalysts 26

**S7 Thermal catalytic CO_2_ hydrogenation performance** 28

**S8 Benchmarking plasma-catalytic CO_2_ hydrogenation** 29

**S9 Supplementary in situ characterization and diagnostic results**

S9.1 In situ plasma-coupled FTIR characterization 30

S9.2 Optical emission spectroscopic diagnostics 30

**S10 Details of DFT calculations** 32

**S1 Supplementary experimental section**

**S1.1 Chemicals**

Ammonium (meta)tungstate hydrate ((NH_4_)_6_H_2_W_12_O_40_·6H_2_O, 99.5%) and Tetraamminepalladium(II) chloride monohydrate (PdCl_2_(NH_3_)_4_, Pd ≥ 43.0%), were purchased from Aladdin Biochemical Technology Co., Ltd. Hydrochloric acid (HCl) was obtained from Sinopharm Chemical Reagent Co., Ltd. Aluminum oxide foam (AF), silicon carbide foam (SF) and nickel foam (NF) were supplied by Guoxiang Environmental Materials Co., Ltd. CO_2_ (> 99 %), H_2_ and Ar (> 99 %) gases were procured from Liquefaction Air Gas Co., Ltd. Unless otherwise specified, all reagents were used as received without further purification. All solutions were prepared using deionized (DI) water.

**S1.2 Definition of key performance metrics**

Each performance metric used in this work is defined as follows. In a dielectric barrier discharge (DBD) plasma reactor, the discharge power (*P*, W) was determined using the Lissajous figure method:

$P=S_{\text{Lissajous}}\times f$ (Eq. 1)

where *S*_Lissajous_ is the area enclosed by the Lissajous figure and *f* is the discharge frequency (kHz).

The specific energy input (SEI, kJ L^-1^) is defined as:

$\text{SEI}=\frac{60\times P}{Q_{\text{gas}}}$ (Eq. 2)

where *Q*_gas_ is the total gas flow rate (mL min^-1^).

The weight hourly space velocity (WHSV, mL g^-1^ h^-1^) is defined as:

$\text{WHSV}=\frac{60\times Q_{\text{gas}}}{m_{\text{c}}}$ (Eq. 3)

where *m_c_* is the mass of the catalyst (g).

The CO_2_ conversion (*C*, %) is defined as:

$C=\frac{c_{\text{in}}-c_{\text{out}}}{c_{\text{in}}}\times100\%$ (Eq. 4)

The selectivity of CO (*S*, %) is calculated as:

$S=\frac{c_{\text{CO}}}{c_{\text{in}}-c_{\text{out}}}\times100\%$ (Eq. 5)

where *c*_in_ and *c*_out_ are the inlet and outlet concentrations of CO_2_ (ppm), respectively. *c*_CO_ is the concentration of CO (ppm). The energy yield (*E*, mol (kWh)^-1^) is defined as:

$E=\frac{60\times Q_{\text{CO}_{2}}\times C\times S}{22.4P}$ (Eq. 6)

where *Q*_CO2_ is the CO_2_ gas flow (mL min^-1^).

**S1.3 Experimental setup**


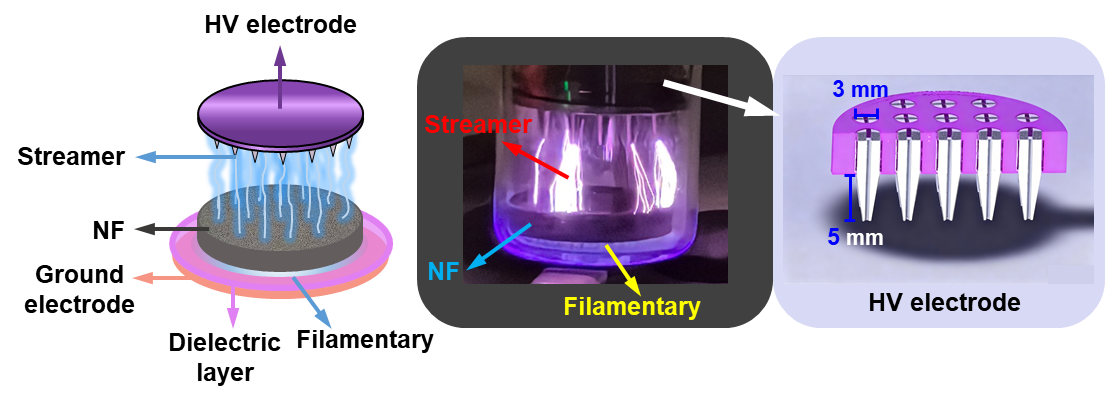


**Figure S1.** Structural of the plasma reactor, photograph of the discharge, and detailed illustration of the high-voltage electrode configuration.


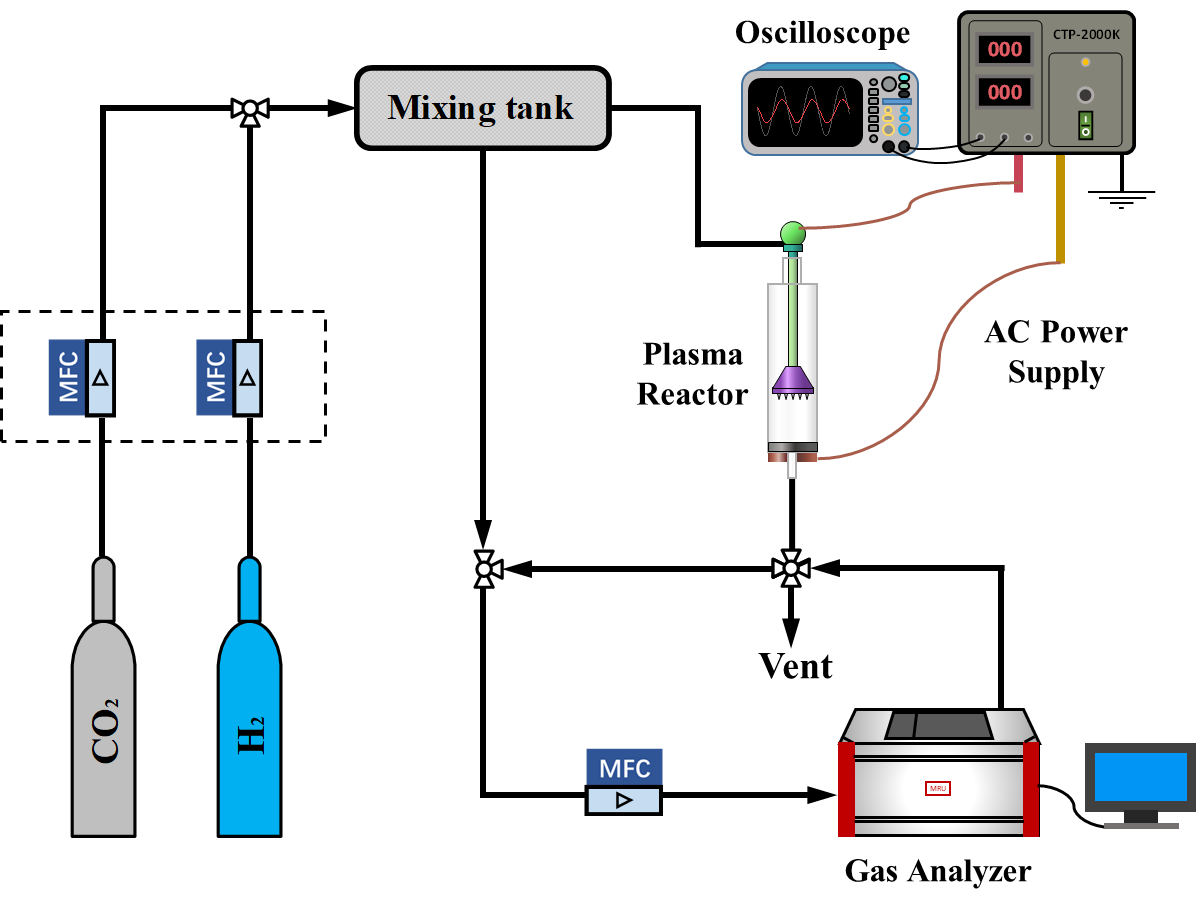


**Figure S2.** Schematic diagram of the experimental setup for plasma-catalytic CO_2_ hydrogenation.

To ensure accurate OES measurement from each discharge zone, a black baffle was used to block emissions from the non-target zone during OES measurements. When measuring the streamer discharge zone, the filamentary discharge zone was shielded, and vice versa. This approach enabled independent, spatially resolved measurement of optical emission spectra from the two discharge zones.


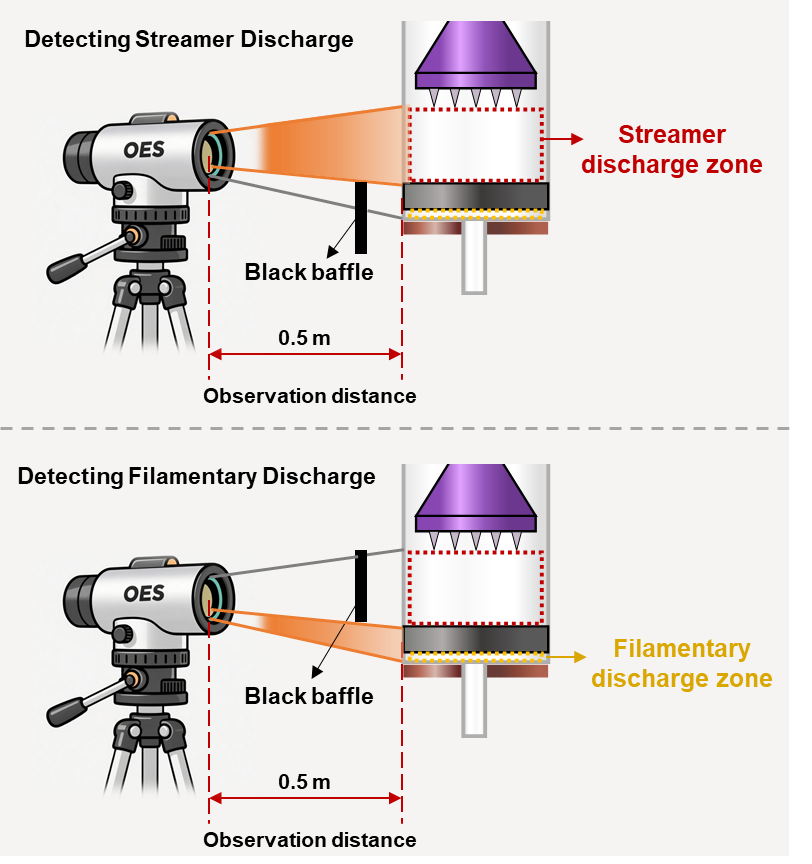


**Figure S3.** Schematic of the OES measurement system. A black baffle was used to isolate optical emissions from different discharge zones (streamer or filamentary) by blocking light from the non-target zone, ensuring accurate and spatially resolved OES measurement.

A schematic of the in situ plasma-coupled transmission Fourier-transform infrared (FTIR) system is shown in **Figure S4**. CO_2_ was introduced at a flow rate of 30 mL min^-1^ for 10 min, followed by H_2_ at the same flow rate to completely displace the CO_2_ in the gas chamber. The plasma was then turned on, and data acquisition was initiated. Prior to entering the FTIR cell, the gas mixture was thoroughly premixed to ensure homogeneity. The custom-designed in situ FTIR cell consists of a rectangular quartz chamber with cylindrical quartz arms allowing unobstructed passage of the IR beam. This configuration facilitates integration of electrodes and catalyst placement. Two circular ports serve as the gas inlet and outlet. A stainless steel 304 needle-type high-voltage electrode and a circular ground electrode are secured to the quartz body using PTFE (polytetrafluoroethylene) tape, allowing flexible adjustment of their relative positions while maintaining gas-tight sealing. The ground electrode is fixed to a quartz support plate with a slotted holder to position the catalyst specimen. The sides of the FTIR cell are sealed with KBr windows mounted with silicone O-rings and screws to ensure optical clarity and airtightness. Plasma was generated using a high-voltage AC power source (Suman, CTP-2000KP) operating at 9.4 kHz and a discharge power of 10–12 W, corresponding to a specific energy input (SEI) of 14–21.3 kJ L^-1^. For IR measurement, 30 mg of catalyst powder (particle size < 50 μm) were thoroughly ground with KBr and pressed into self-supporting pellets (2 mm thickness) under a pressure of 5 MPa. The prepared specimen was mounted onto the holder within the FTIR cell and pretreated under flowing H_2_ until the flow conditions stabilized. A plasma pretreatment was then applied for 1 h. After plasma activation, Ar was purged through the cell for 15 min, and the background spectra were collected. Plasma-catalytic CO_2_ hydrogenation was carried out at ambient temperature (20 °C) and atmospheric pressure (1 atm). Spectral data were collected and analyzed using the OMNIC software suite to monitor the evolution of adsorbed species during plasma exposure.


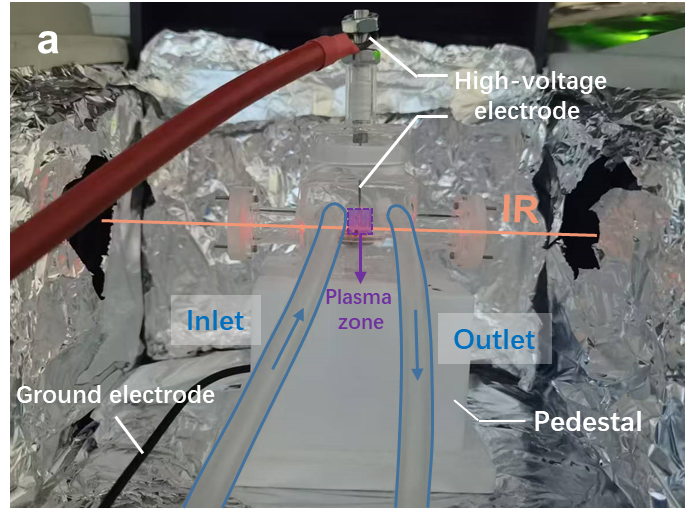


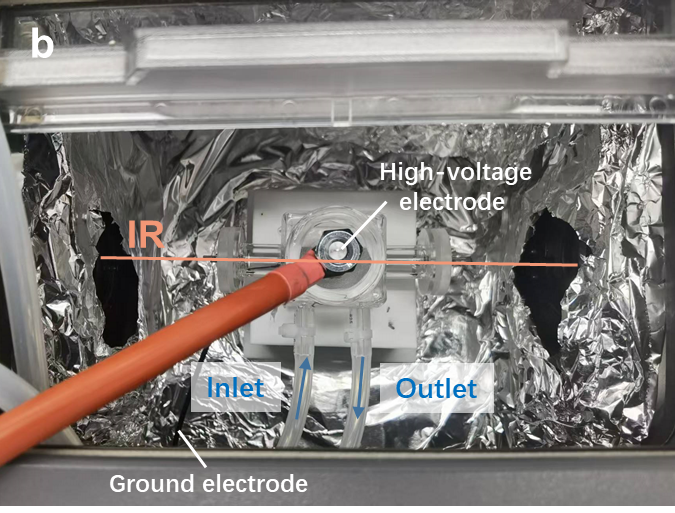


**Figure S4.** (a) Side view and (b) top view of the in situ plasma-coupled FTIR system.

**S1.4 Computational details**

All spin-polarized density functional theory (DFT) calculations were conducted using the Vienna ab initio simulation package (VASP). Van der Waals interactions were accounted for using the D3 correction scheme (DFT-D3). The exchange-correlation functional was treated with the generalized gradient approximation (GGA) of Perdew–Burke–Ernzerhof (PBE). Core–valence interactions were described using the projector-augmented wave (PAW) method. Electron smearing was applied using the Gaussian smearing method with a width of 0.05 eV, and the plane-wave energy cutoff was set to 500 eV. Structural optimizations were performed by minimizing the forces on each ion using the conjugate gradient algorithm until forces were below 0.02 eV Å^-1^. Self-consistent calculations employed an energy convergence criterion of 10^-5^ eV. The Monkhorst–Pack k-point mesh was used for Brillouin-zone integration with a k-point separation of 0.04 Å^−1^.

Reaction energies (Δ*E*) and activation energies (*E*_a_) were calculated using the Born–Oppenheimer energy differences between the initial state (IS), transition state (TS), and final state (FS):

Δ*E* = *E*_FS_*－E*_IS_ (Eq. 7)

*E*_a_ = *E*_TS_*－E*_IS_ (Eq. 8)

where *E*_IS_, *E*_TS_, and *E*_FS_ are the energies of the IS, TS, and FS, respectively. The adsorption energy (*E*_ads_) was calculated as:

*E*_ads_ = *E*_total_－*E*_substrate_－*E*_adsorbate_ (Eq. 9)

where *E*_total_, *E*_substrate_, and *E*_adsorbate_ are the total energies of the combined system, the substrate, and the adsorbate, respectively.

The charge density difference was obtained using:

Δ*ρ* = *ρ*_AB_ − *ρ*_A_ − *ρ*_B_ (Eq. 10)

where Δ*ρ* represents the charge density of the total system, and *ρ*_A_ and *ρ*_B_ correspond to the charge densities of fragments A and B, respectively.

The free energy of adsorbates was calculated from harmonic vibrational frequencies hvi using VASPKIT (v.1.2.0) according to the following formulas:

*F* = *U* − *T* × S (Eq. 11)

*U = E*_DFT_ + *E*_ZPE_ + *U(T)* (Eq. 12)

 (Eq. 13)

 (Eq. 14)

where *F* is the free energy, *U* is the internal energy, *T* is the temperature, *S* is the entrop, *E*_DFT_ is the DFT energy, *E*_ZPE_ is the zero-point energy, *U(T)* is the thermal correction, and *v*_i_ are the vibrational frequencies. Here, *h* is Planck’s constant, and *k* is Boltzmann’s constant.

**S2 Supplementary catalyst characterization (ICP-OES, SEM, BET and CO-TPD)**

**S2.1 ICP-OES analysis of the catalysts**

**Table S1.** Actual loadings of WO_3-x_/NF and Pd-WO_3-x_/NF catalysts.

| Catalyst | W loading (wt.%) | Pd loading (wt.%) |
| --- | --- | --- |
| WO_3-x_/NF | 15.76 | 0 |
| Pd-WO_3-x_/NF | 14.14 | 0.99 |

**S2.2 SEM analysis of catalyst morphology**


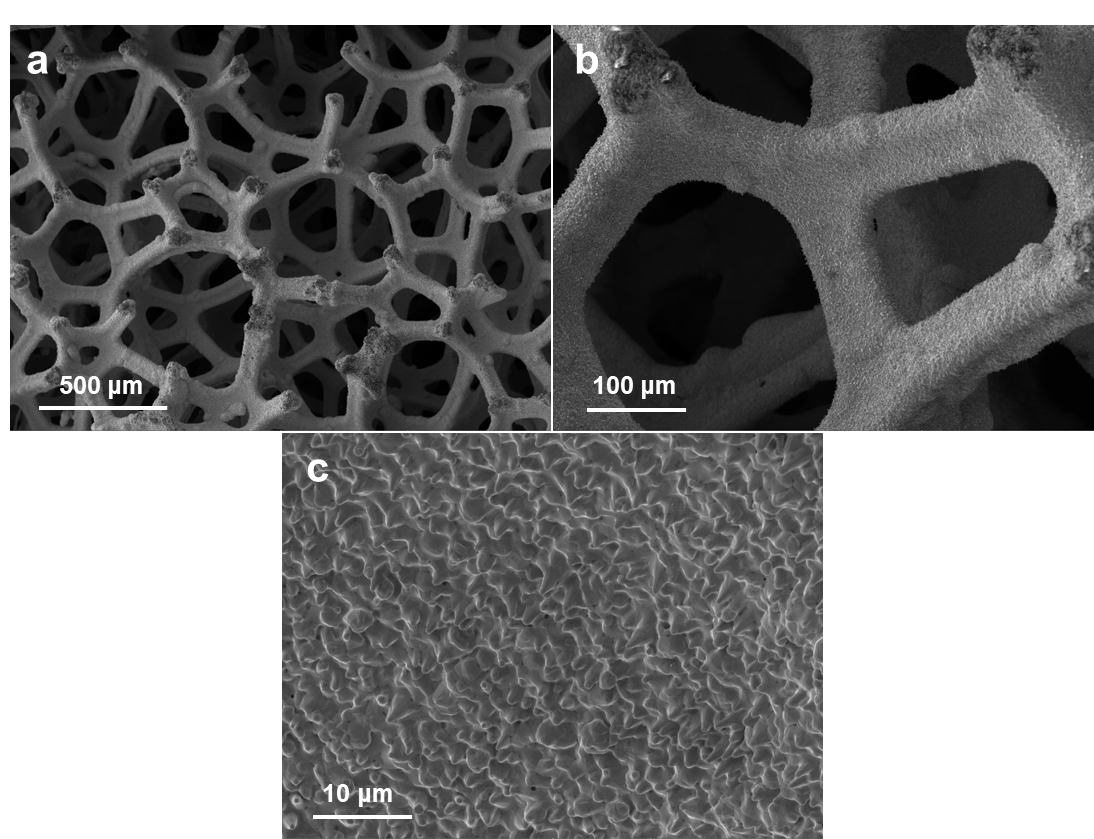


**Figure S5.** SEM images of bare NF.


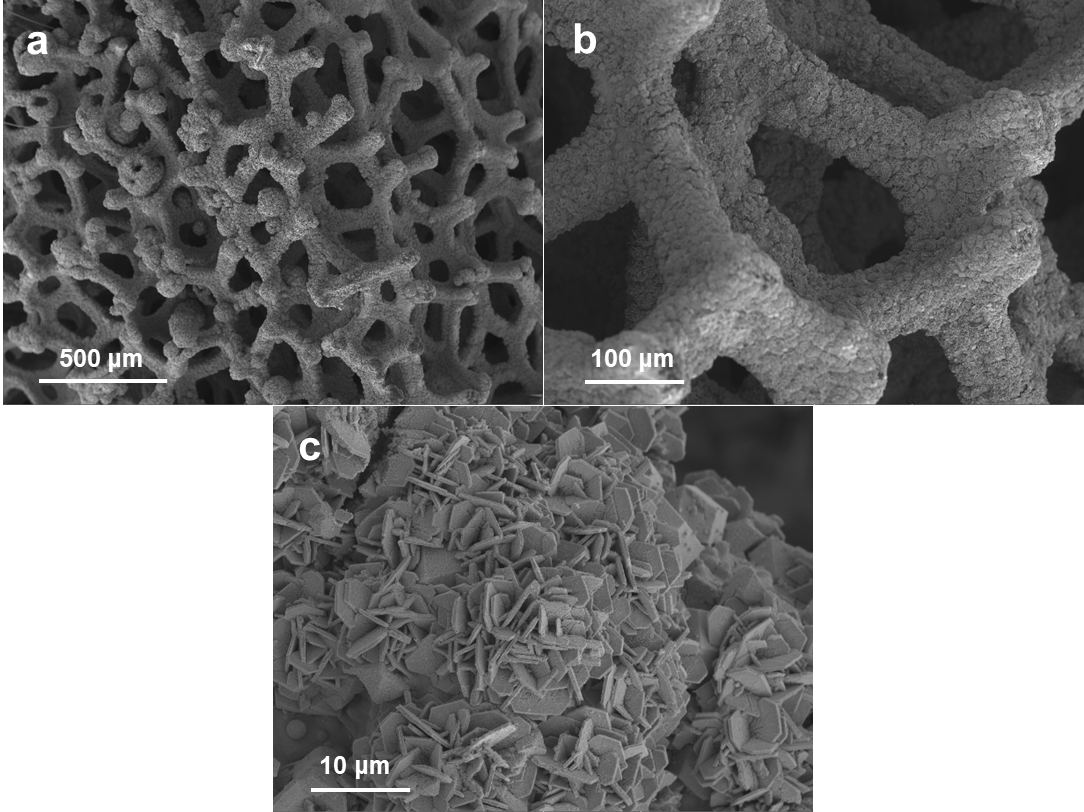


**Figure S6.** SEM images of WO_3-x_/NF.


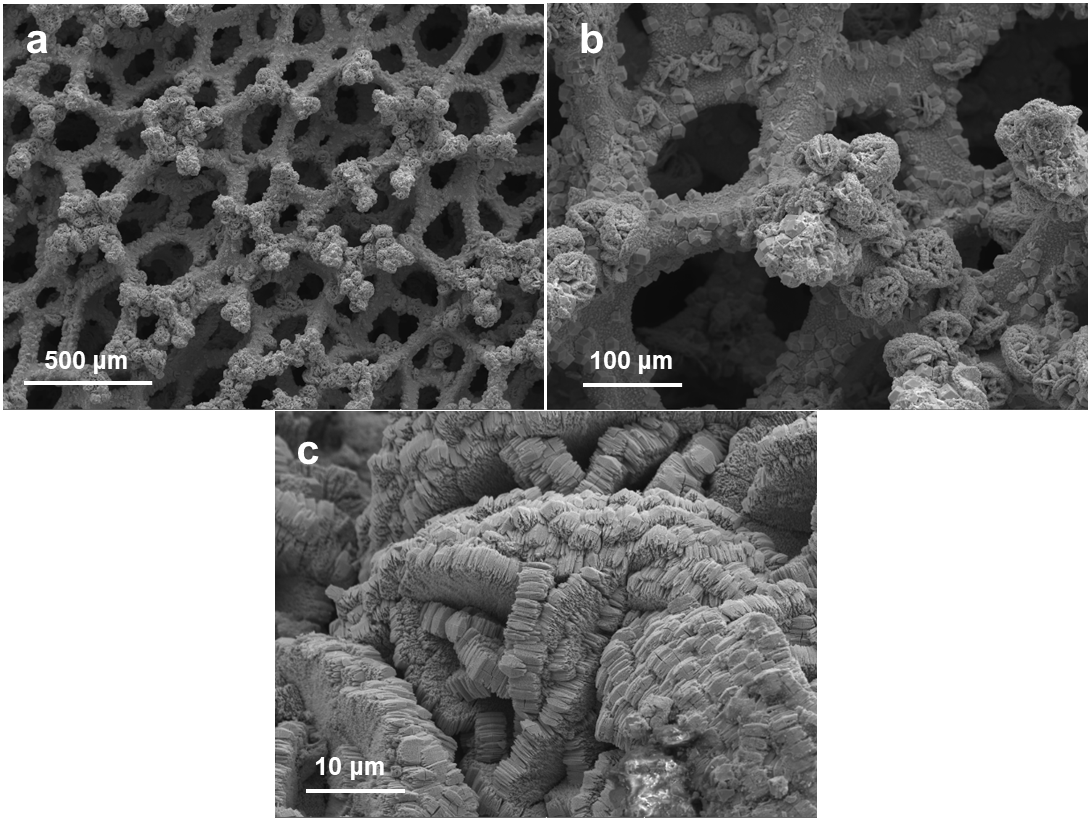


**Figure S7.** SEM images of Pd-WO_3-x_/NF.


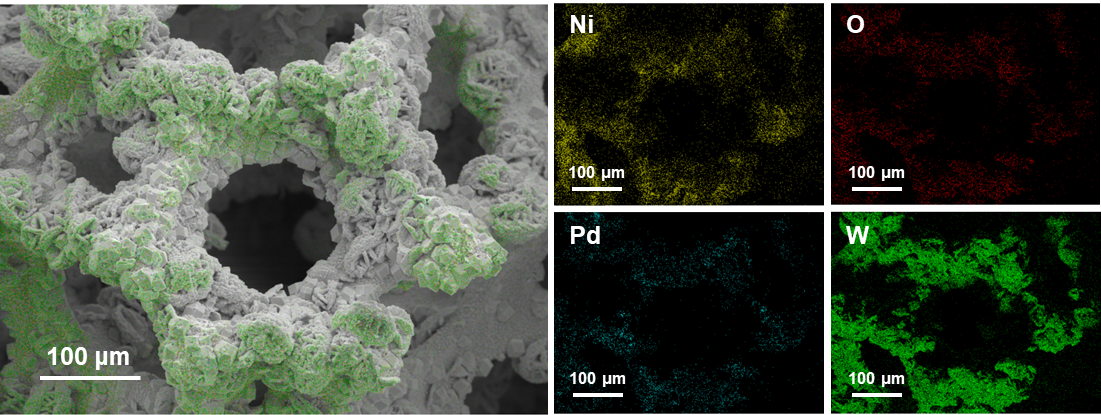


**Figure S8.** SEM-EDS elemental mapping of Pd-WO_3-x_/NF.

**S2.3 BET analysis of the catalysts**


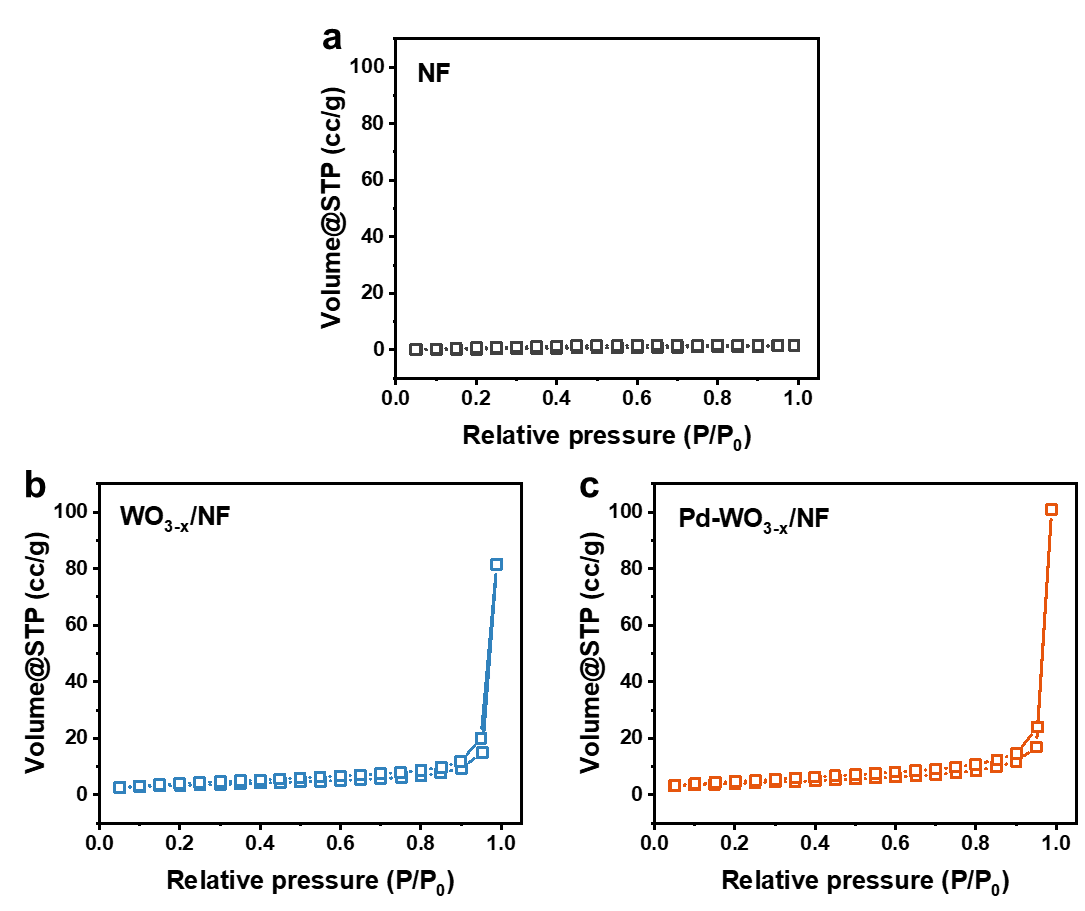


**Figure S9.** N_2_ adsorption and desorption curves of (a) NF, (b) WO_3-x_/NF and (c) Pd-WO_3-x_/NF.

**S2.4 CO-TPD analysis**

CO-TPD measurements were performed to quantify accessible metal sites, revealing that Pd-WO_3-x_/NF exhibits 2.8-fold higher CO uptake than WO_3-x_/NF, confirming enhanced active site exposure. Notably, the spent catalyst retains 75.5% of its initial CO adsorption capacity (213.5 μmol g^-1^ for fresh vs. 161.3 μmol g^-1^ for spent) without significant peak broadening (**Figure S10**), suggesting that the number of accessible Pd active sites decreases moderately without severe aggregation.


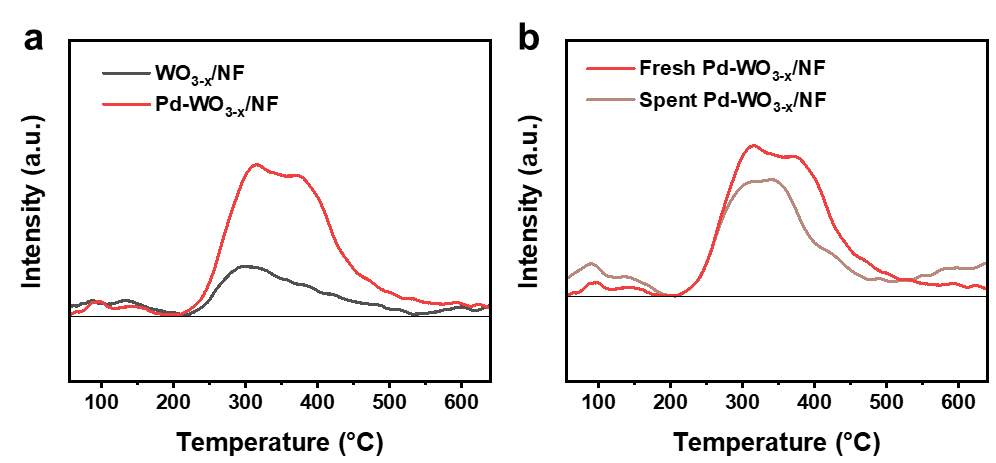


**Figure S10.** CO-TPD profiles of (a) WO_3-x_/NF and Pd-WO_3-x_/NF, and (b) fresh and spent Pd-WO_3-x_/NF.

**Table S2.** CO-TPD analysis of the catalysts.

| Catalyst | WO_3-x_/NF | Pd-WO_3-x_/NF | |
| --- | --- | --- | --- |
| CO-TPD (μmol g^-1^) | 75.2 | 213.5 (fresh) | 161.3 (spent) |

**S3 Plasma electrical and optical diagnostics**

**S3.1 Electrical signal analysis**

In the absence of the NF substrate (plasma-only), the current exhibited a sinusoidal waveform with low-intensity multipulse superposition (*I*_p_ = 39.7 mA), indicating that the discharge was primarily composed of dispersed weak pulses. Upon the introduction of the NF substrate, the current waveform evolved into a high-intensity pulsed pattern (*I*_p_ = 99.4 mA), suggesting that NF promoted plasma strengthening and accelerated charge transfer. After loading WO_3-x_ onto NF, the peak current slightly decreased to 87.9 mA (**Figure S11**). This reduction can be ascribed to the semiconducting nature of WO_3-x_, where partial electronic conductivity sustains steady-state charge transport (elevated *I*_RMS_), while interfacial polarization suppresses transient discharge intensity (reduced *I*_p_). Further incorporation of Pd nanoparticles (WO_3-x_/NF) led to a pronounced increase in the peak current (*I*_p_ = 106.6 mA), confirming that Pd enhances the transient discharge capability through improved surface charge dynamics.

As shown in **Figure S12**, the peak voltage decreased from 17.4 to 13.8 kV upon introducing the NF substrate, which is consistent with the evolution of the current waveforms (**Figure S11**). This voltage reduction reflects the enhanced discharge efficiency and localization induced by NF, facilitating more concentrated plasma–surface interactions.


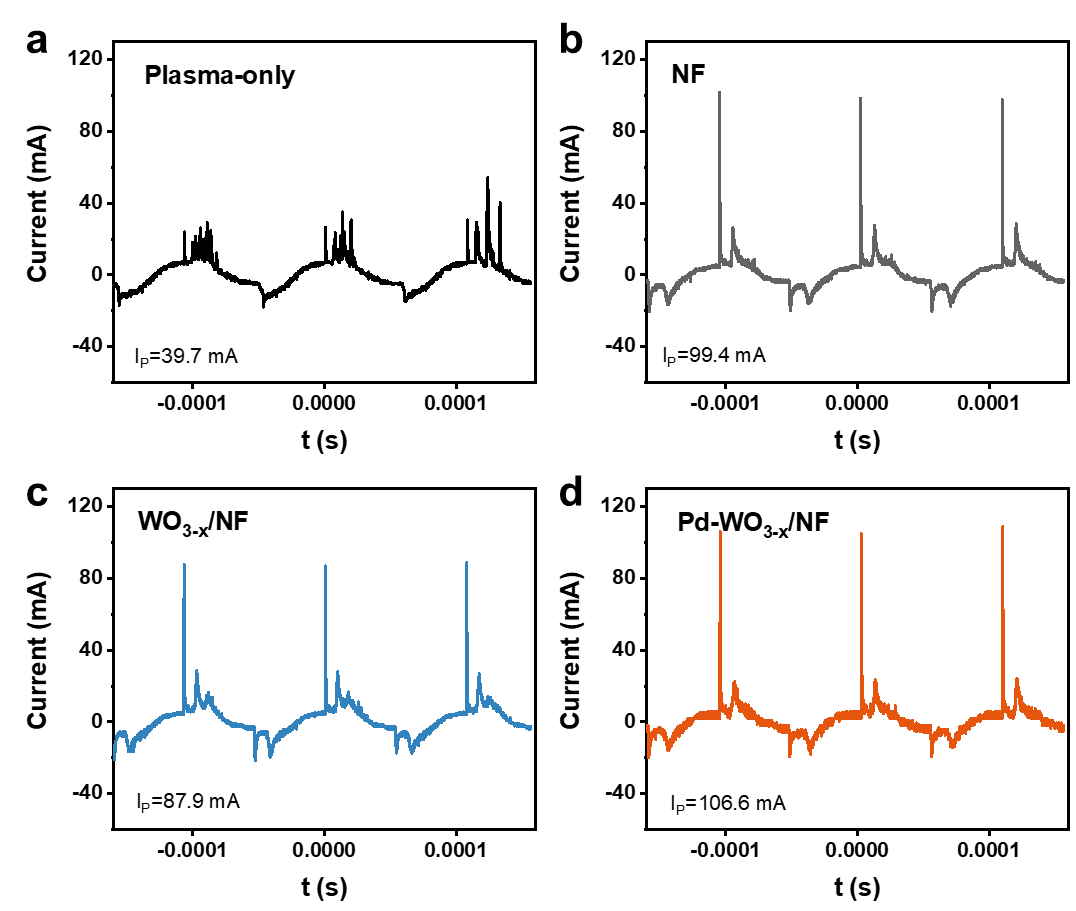


**Figure S11.** Current waveforms of (a) plasma-only, (b) NF, (c) WO_3-x_/NF and (d) Pd-WO_3-x_/NF.


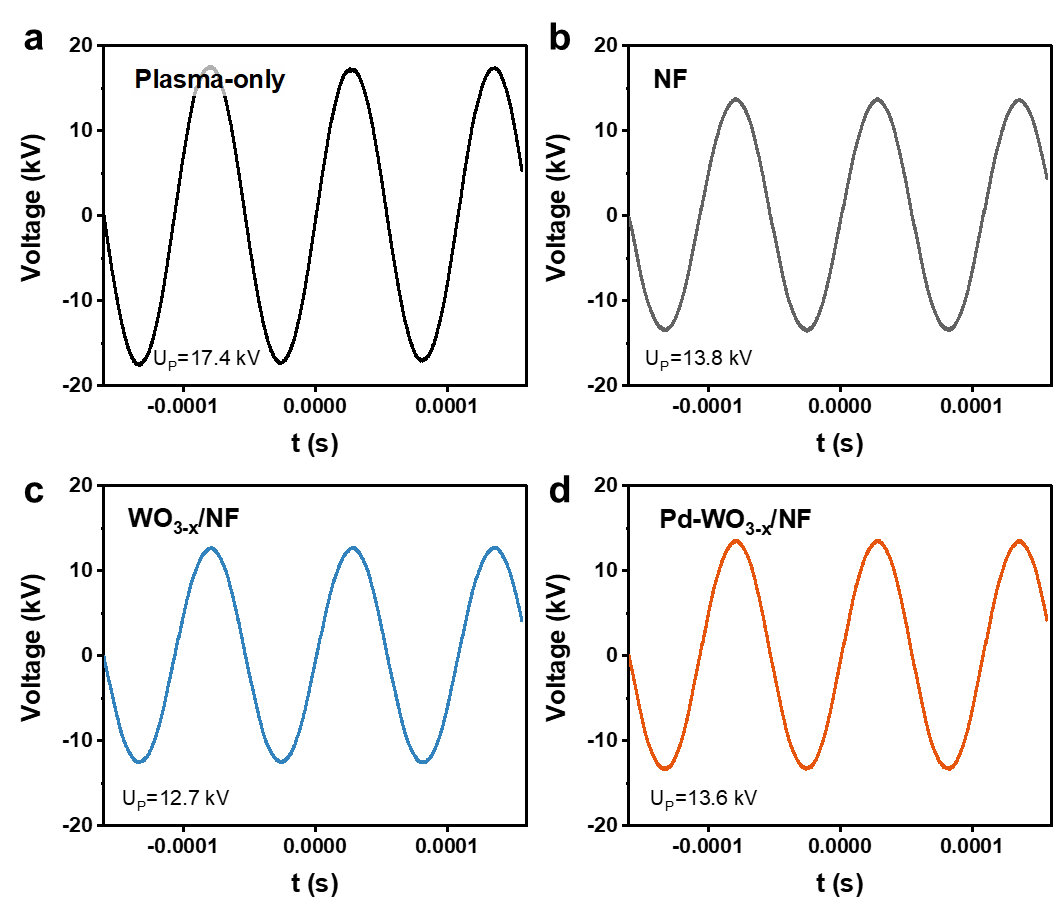


**Figure S12.** Voltage waveforms of (a) plasma-only, (b) NF, (c) WO_3-x_/NF and (d) Pd-WO_3-x_/NF.

**S3.2 Optical emission spectroscopic diagnostics**

**Figure S13.** In situ emission spectra of CO_2_/H_2_ plasma in the streamer discharge zone over NF, WO_3-x_/NF and Pd-WO_3-x_/NF.

**S4 Supplementary catalyst characterization (HRTEM, XRD, H_2_-TPR and CO_2_-TPD)**

**S4.1 HRTEM analysis of the catalysts**

**Figure S14** shows the HRTEM images and corresponding particle size distributions of fresh and spent Pd-WO_3-x_/NF. The average Pd particle size remains nearly unchanged before and after the reaction (2.3 nm for fresh and 2.1 nm for spent), confirming that no significant aggregation or sintering of Pd NPs occurs during the plasma-catalytic RWGS reaction.

**
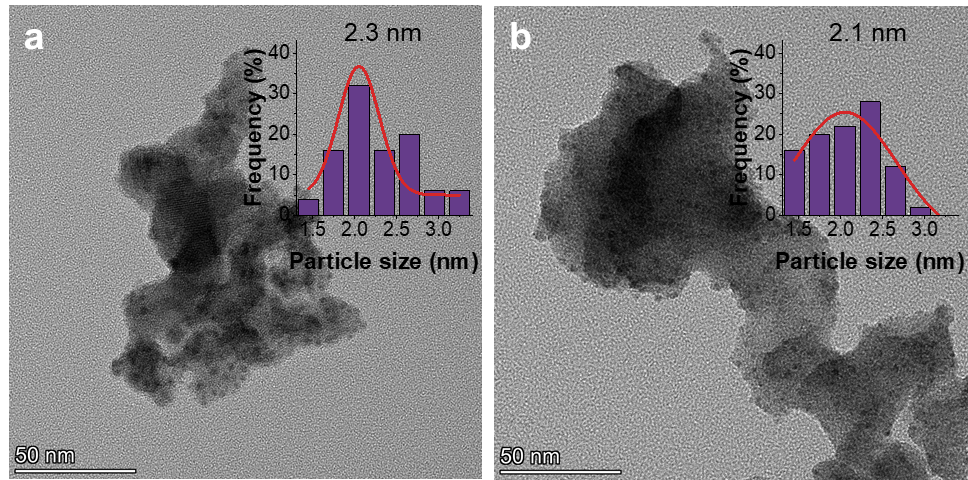
**

**Figure S14.** HRTEM images of (a) fresh and (b) spent Pd-WO_3-x_/NF, together with corresponding particle size distributions (inset) and average particle sizes (on top).

**S4.2 XRD analysis**

**Figure S15.** XRD patterns of WO_3-x_/NF and Pd-WO_3-x_/NF.

**S4.3 H_2_-TPR analysis**

**Figure S16.** H_2_-TPR profiles of WO_3-x_/NF and Pd-WO_3-x_/NF.

**Table S3.** Desorption peak areas and hydrogen consumption of the catalysts determined from H_2_-TPR.

| Catalyst | Desorption area of H_2_ | H_2_ consumption (μmol g^-1^) |
| --- | --- | --- |
| WO_3-x_/NF | 24502100 | 2017.7 |
| Pd-WO_3-x_/NF | 21614000 | 1779.9 |

**S4.4 CO_2_-TPD analysis**

**Figure S17.** CO_2_-TPD profiles of WO_3-x_/NF and Pd-WO_3-x_/NF.

**Table S4.** Desorption peak areas and total alkalinity of the catalysts determined from CO_2_-TPD.

| Catalyst | Desorption area of CO_2_ | Total alkalinity (mmol g^-1^) |
| --- | --- | --- |
| WO_3-x_/NF | 1516390 | 0.121 |
| Pd-WO_3-x_/NF | 1750750 | 0.140 |

**S5 Supplementary plasma-catalytic performance**

**S5.1 CO_2_ conversion using different catalysts and substrates**

The optimal CO_2_ conversion is achieved when the SEI is 33.6 kJ L^-1^ (**Figure S18**). Therefore, we adhered to standardizing at 33.6 kJ L^-1^ for comparative studies. By separating discharge zones in the plasma reactor, the notable dielectric properties of NF were exploited to enhance streamer discharges, which are not achievable with low dielectric substrates such as alumina (Al_2_O_3_) or silicon carbide (SiC). The enhancement in streamer discharge performance of NF substrates was quantitatively compared with alumina foam (AF) and silicon carbide foam (SF) substrates (**Figure S19**). In the absence of streamer discharge (i.e., in a packed-bed configuration), AF and SF slightly outperformed NF due to their inherent conductivity, which leads to partial quenching of the plasma.

To assess the intrinsic catalytic contribution of bare NF, experiments were performed under conditions dominated by filamentary discharge (discharge gap = 0 mm). Under these conditions, CO_2_ conversion for bare NF was 14.7% (**Figure S19**). For validation, an additional plasma-only experiment (without NF) was conducted under identical conditions, yielding a CO_2_ conversion of 15.2%, which is comparable to that of bare NF. This comparison demonstrates that, when excluding the enhancement from streamer discharge, the direct catalytic effect of NF is negligible.

**Figure S18.** Effect of SEI on CO_2_ conversion over NF, WO_3-x_/NF and Pd-WO_3-x_/NF. WHSV = 10,870 mL g^-1^ h^-1^.

**Figure S19.** Effect of discharge gap on CO_2_ conversion over substrates with different dielectric properties: Al_2_O_3_ foam (AF), SiC foam (SF), and NF. WHSV = 10,870 mL g^-1^ h^-1^, SEI = 33.6 kJ L^-1^.

**S5.2 Effect of discharge gap on CO_2_ conversion**

**Figure S20** compares the CO_2_ conversion over NF, WO_3-x_/NF and Pd-WO_3-x_/NF at different discharge gaps (0–15 mm). When the discharge gap is 0 mm, the streamer discharge zone is absent. CO_2_ conversion follows the order: NF (15.7%) <WO_3-x_/NF (18.3%) <Pd-WO_3-x_/NF (26.1%). As the gap increases to 15 mm (corresponding to the streamer discharge zone), CO_2_ conversion significantly increases, reaching 37.8% (NF), 41.8% (WO_3-x_/NF) and 54.9% (Pd-WO_3-x_/NF). These results suggest that a wider discharge gap generates the streamer discharge zone, which enhances the synergistic effect of the plasma catalysis by expanding the high-energy, electron-rich region. Pd-WO_3-x_/NF consistently outperforms the other samples, highlighting the critical role of the Pd-WO_3-x_ interfacial site in plasma-catalytic CO_2_ hydrogenation.

**Figure S20.** Effect of discharge gap on CO_2_ conversion over NF, WO_3-x_/NF and Pd-WO_3-x_/NF. WHSV = 10,870 mL g^-1^ h^-1^, SEI = 33.6 kJ L^-1^.

**S5.3 Effect of WHSV on CO_2_ conversion**

An increase in WHSV leads to a decrease in the specific energy input (SEI), reflecting a reduction in the energy delivered to the plasma system. Consequently, CO_2_ conversion decreases with increasing WHSV. For example, when WHSV was increased from 10,870 mL g^-1^ h^-1^ to 100,000 mL g^-1^ h^-1^, the SEI dropped significantly from 36.6 kJ L^-1^ to 4.0 kJ L^-1^, resulting in a decrease in CO_2_ conversion from 56.6% to 38.4% (Figure S21).

**Figure S21.** Effect of WHSV and SEI on CO_2_ conversion over WO_3-x_/NF and Pd-WO_3-x_/NF.

**
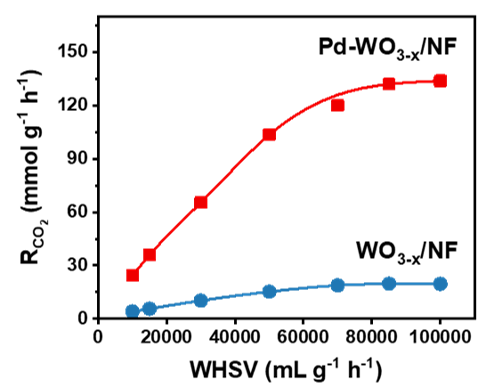
**

**Figure S22.** CO_2_ reaction rate (R_CO2_) over WO_3-x_/NF and Pd-WO_3-x_/NF as a function of WHSV.

**S5.4 Effect of H_2_/CO_2_ ratio and discharge frequency on plasma-catalytic CO_2_ hydrogenation**

The effect of H_2_/CO_2_ mole ratio and discharge frequency on the performance of plasma-catalytic CO_2_ hydrogenation was systematically investigated. As shown in **Figure S23**, CO_2_ conversion increased from 25.4 to 54.9% as the H_2_/CO_2_ ratio increased from 0 to 1.5, indicating that H_2_ plays a key role in the CO_2_ reduction reaction. However, CO_2_ conversion decreased significantly at higher H_2_ concentrations, dropping to 30.6% when the H_2_/CO_2_ ratio reached 3. This decline is attributed to excessive H_2_ competing for plasma energy, leaving insufficient energy for CO_2_ activation, while accumulation of H radicals may promote side reactions such as H_2_ recombination.

To optimize system parameters, the effect of discharge frequency (8.2 –10.6 kHz) was examined at a fixed SEI of 33.6 kJ L^-1^ and WHSV of 10,870 mL g^-1^ h^-1^. As shown in **Figure S24**, excessively high frequency (e.g., 10.6 kHz) limits the energy input per cycle, reducing the vibrational excitation efficiency of CO_2_ and lowering conversion from 54.9% to 41.3%. Conversely, too low a frequency (e.g., 8.2 kHz) results in energy dissipation due to increased dielectric losses. An optimal frequency of 9.4 kHz balances electron excitation efficiency and energy transfer, achieving the maximum CO_2_ conversion.

**Figure S23.** Effect of H_2_/CO_2_ molar ratio on CO_2_ conversion over Pd-WO_3-x_/NF. SEI = 36.6 kJ L^-1^, WHSV = 10,870 mL g^-1^ h^-1^.

**Figure S24.** Effect of discharge frequency on CO_2_ conversion over NF, WO_3-x_/NF and Pd-WO_3-x_/NF. SEI = 36.6 kJ L^-1^, WHSV = 10,870 mL g^-1^ h^-1^.

**S6 Supplementary** **characterization of spent Pd-WO_3-x_/NF catalysts**

**S6.1 XPS analysis of fresh and spent catalysts**

XPS analysis reveals that the surface chemical state of the catalyst evolves after the reaction (**Figure S25**). The binding energy of W 4f decreased from 32.6 to 32.3 eV, accompanied by a reduction in peak intensity. Notably, the intensity ratio of W^4+^/W^5+^ decreased from 1.17 for the fresh catalyst to 0.85 for the spent catalyst. This suggests that, although continuous electron injection in the plasma environment enriches electron density at tungsten sites, a portion of W^4+^ is still oxidized to W^5+^/W^6+^. The O_def_/O_lat_ ratio in the O 1s spectrum decreased from 0.43 to 0.37, consistent with partial oxidation of oxygen vacancies (OVs) observed in O_2_-TPO measurements, confirming dynamic consumption and regeneration of OVs during the reaction. The Pd 3d peak position remains unchanged, indicating that Pd NPs retain their metallic state, ensuring sustained H_2_ dissociation and electron transfer capability. In the C 1s spectrum, the peak at 284.8 eV (amorphous carbon) increased 4–5 times in intensity, and new peaks at 286.5 eV (C–O) and 288.8 eV (O–C=O) appear, reflecting accumulation of reaction intermediates such as *CO and carbonate species on the surface. Nevertheless, SEM and HRTEM images show no significant carbon deposition or structural degradation (**Figures S27–S28**), suggesting that some adsorbed species are removed via plasma-induced transient desorption, thereby avoiding blockage of active sites. The dynamic equilibrium of surface chemical states, including limited consumption of OVs, stable electron transfer at the Pd–W interface, and controlled adsorption of intermediates, explains the catalyst’s ability to maintain 90.2% activity over a 100 h stability test.


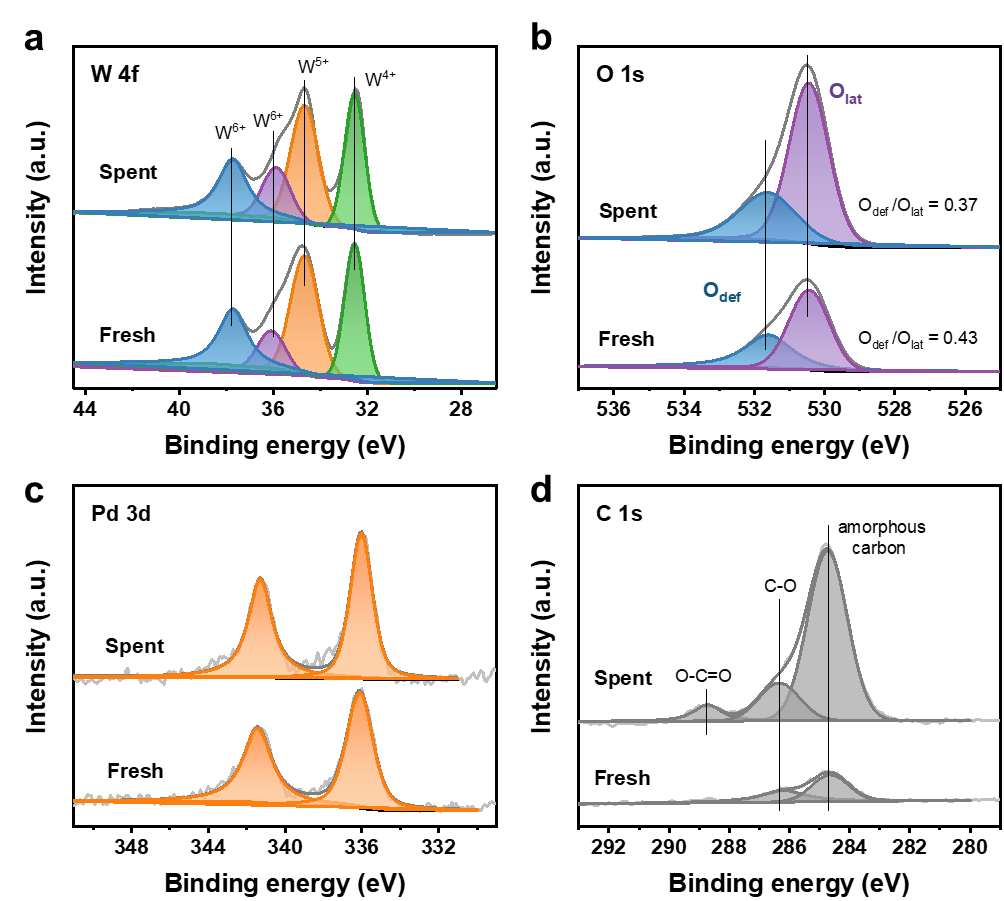


**Figure S25.** XPS spectra of (a) fresh and (b) spent (after 100 h catalyst stability test, the same below) Pd-WO_3-x_/NF. Catalyst stability test conditions: SEI = 36.6 kJ L^-1^, WHSV=10,870 mL g^-1^ h^-1^.

**S6.2 O_2_-TPO analysis of fresh and spent catalysts**

O_2_-TPO analysis of the spent catalyst (**Figure S26**) revealed a new oxygen consumption peak at 358 °C in addition to the original 424 °C peak observed in the fresh catalyst, accompanied by a decrease in total oxygen uptake from 12.43 to 8.53 mmol g^-1^. These changes indicate partial oxidation of metastable oxygen vacancies (OVs) into stabilized lattice oxygen species during prolonged plasma exposure. This process mitigates surface over-reduction, which is critical for preserving active tungsten sites and maintaining interfacial charge balance.

**Figure S26.** O_2_-TPO profiles of (a) fresh and (b) spent Pd-WO_3-x_/NF. Catalyst stability test conditions: SEI = 36.6 kJ L^-1^, WHSV=10,870 mL g^-1^ h^-1^.

**S6.3 SEM and HRTEM characterization of fresh and spent catalysts**

Post-reaction characterization of the catalyst using SEM, HRTEM, and XRD (**Figures S27–S29**) revealed no significant structural degradation or phase changes, demonstrating the excellent stability of Pd-WO_3-x_/NF under plasma reaction conditions.


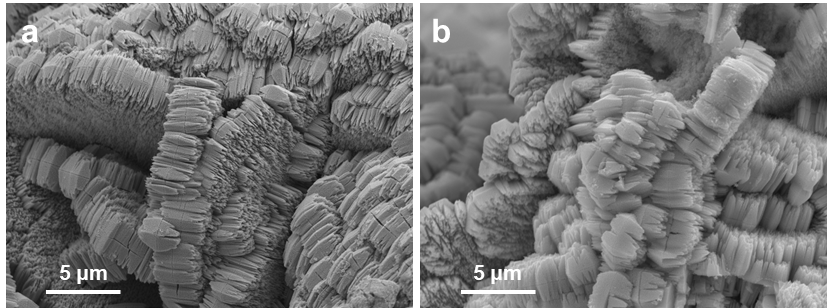


**Figure S27.** SEM images of (a) fresh and (b) spent Pd-WO_3-x_/NF. Catalyst stability test conditions: SEI = 36.6 kJ L^-1^, WHSV=10,870 mL g^-1^ h^-1^.


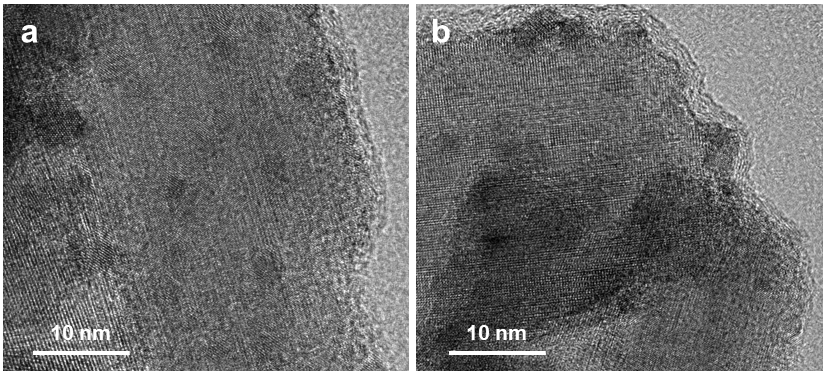


**Figure S28.** HRTEM images of (a) fresh and (b) spent Pd-WO_3-x_/NF. Catalyst stability test conditions: SEI = 36.6 kJ L^-1^, WHSV=10,870 mL g^-1^ h^-1^.

**S6.4 XRD analysis of fresh and spent catalysts**

**Figure S29.** XRD patterns of fresh and spent Pd-WO_3-x_/NF. Catalyst stability test conditions: SEI = 36.6 kJ L^-1^, WHSV=10,870 mL g^-1^ h^-1^.

**S6.5 EPR analysis of fresh and spent catalysts**

EPR analysis shows that the signal intensity of the spent catalyst decreased by 8%, indicating partial oxidation of oxygen vacancies, while highlighting the stabilizing effect of Pd in preserving most of the active sites.

**Figure S30.** EPR analyses of fresh and spent Pd-WO_3-x_/NF catalysts (after 100 h stability test). Test conditions: SEI = 36.6 kJ L^-1^, WHSV=10,870 mL g^-1^ h^-1^.

**S6.6 O_2_-TPD analysis of fresh and spent catalysts**

O_2_-TPD analysis was conducted to investigate the evolution of oxygen species in fresh and spent catalysts. The fresh catalyst exhibits a prominent peak at 273 °C, attributed to active oxygen adsorbed on oxygen vacancies, along with triplet peaks at 495, 521, and 543 °C corresponding to lattice oxygen in distinct microenvironments. After reaction, the spent catalyst shows a slight decrease in the 273 °C peak intensity, with quantitative analysis revealing oxygen desorption amounts of 0.23 mmol g⁻¹ for the fresh catalyst and 0.21 mmol g⁻¹ for the spent catalyst. These results indicate that, although some oxygen vacancies were partially oxidized, the majority were preserved due to the stabilizing effect of Pd. Additionally, a broadened peak at 538 °C in the spent catalyst suggests lattice oxygen reconstruction and fusion, leading to the merging of the triplet desorption peaks into a single broader feature.

**Figure S31.** O_2_-TPD profiles of fresh and spent Pd-WO_3-x_/NF catalysts (after 100 h stability test). Test conditions: SEI = 36.6 kJ L^-1^, WHSV=10,870 mL g^-1^ h^-1^.

**S7 Thermal catalytic CO_2_ hydrogenation performance**


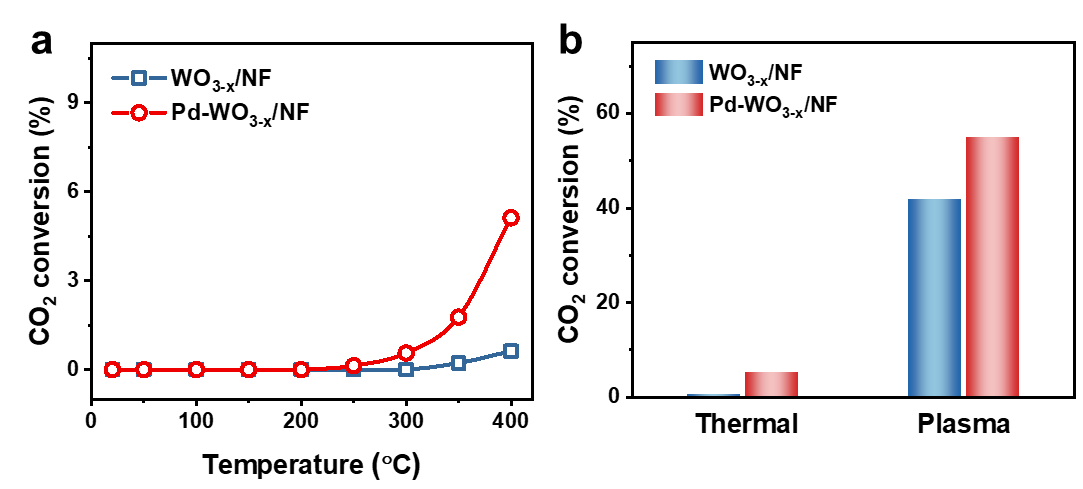


**Figure S32.** (a) Effect of reaction temperature on CO_2_ conversion in thermal catalytic CO_2_ hydrogenation over WO_3-x_/NF and Pd-WO_3-x_/NF. (b) CO_2_ conversion under thermal catalysis at 400 ℃ and plasma catalysis at a discharge power of 28 W. WHSV = 10,870 mL g^-1^ h^-1^.

**S8 Benchmarking plasma-catalytic CO_2_ hydrogenation**

**Table S5.** Benchmarking of plasma-catalytic CO_2_ hydrogenation reported in the literature and in this work.

| Catalyst | Reactor | SEI  (kJ L^-1^) | CO_2_ conversion (%) | Energy yield  (mol kWh^-1^) | CO_2_ reaction rate (mmol g^-1^ h^-1^) | Catalyst stability (h) | Ref. |
| --- | --- | --- | --- | --- | --- | --- | --- |
| Au/CeO_2_ | DBD | 9 | 25.5 | 0.455 | 68.3 | 5 | (1) |
| 5Fe5Ce/Al_2_O_3_ |  | 22.5 | 24.5 | 1.75 | 16.41 | - | (2) |
| OV–In_2_O_3_ |  | 109.8 | 60.8 | 0.89 | 217.15 | 8.3 | (3) |
| Pd/ZnO |  | 30 | 36.7 | 0.475 | 19.66 | 6 | (4) |
| Cu/Ti_3_C_2_T_x_ |  | 120 | 31.9 | 0.401 | 60.24 | 10 | (5) |
| 13.1Pd-2.5Cu |  | 14.8 | 38 | 0.132 | 20.36 | 24 | (6) |
| Ce_x_Zr_1−x_O_2_ OC |  | 7.87 | 84 | 0.815 | 56.25 | - | (7) |
| Co_x_O_y_/MgO |  | 21.4 | 35 | 0.066 | 13.13 | 4.5 | (8) |
| CeO_2_-NiO |  | 106.7 | 19 | 0.082 | 5.73 | - | (9) |
| LNO-800 |  | 180 | ~58 | 0.14 | 125 | - | (10) |
| Ni/γ-Al_2_O_3_ |  | 80 | 26.3 | 0.528 | 0.384 | - | (11) |
| Ni/Al_2_O_3_ |  | 33.82 | 67.8 | 0.644 | 62.84 | - | (12) |
| La_0.9_Sr_0.1_Ni_0.5_Fe_0.5_O_3+δ_ |  | 267 | ~60 | 0.163 | 160.7 | 100 | (13) |
| Pd-WO_3-x_/NF | Without streamer discharge | 14.4-33.6 | 26.1 | 0.74 | 153.0 | 46 | This work |
|  | With streamer discharge |  | 54.9 | 1.87 | 441.1 |  |  |

**S9** **Supplementary in situ characterization and diagnostic results**

**S9.1 *In situ* plasma-coupled FTIR characterization**


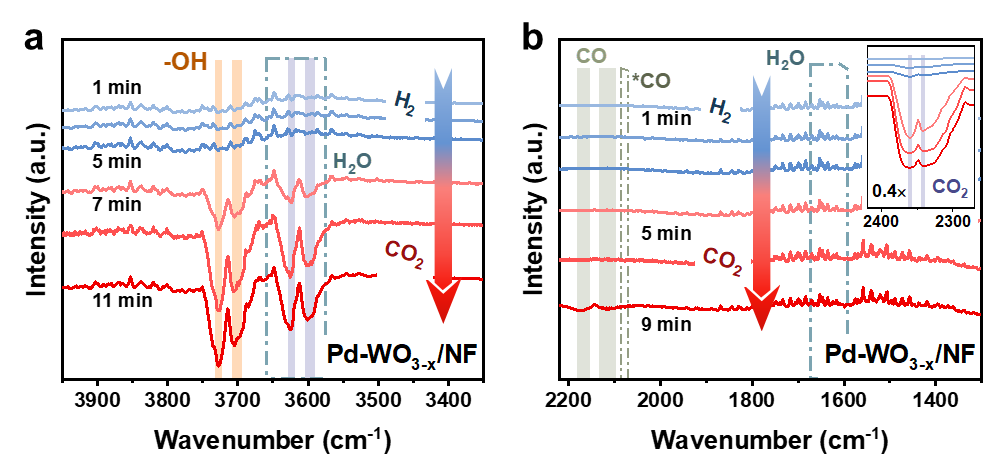


**Figure S33.** In situ FTIR spectra of surface species on Pd-WO_3-x_/NF during alternating H_2_ and CO_2_ exposure at a gas flow rate of 30 mL min^-1^.

**S9.2 Optical emission spectroscopic diagnostics**

**Figure S34.** Emission spectra of CO_2_/H_2_ plasma under plasma-only (gray), filamentary discharge (orange) and streamer discharge (purple) conditions using bare nickel foam (NF). The distinct emission spectra highlight differences in plasma species generated in each discharge zone.

**Figure S35.** Emission spectra of CO_2_/H_2_ plasma in the filamentary discharge zone over NF, WO_3-x_/NF and Pd-WO_3-x_/NF.

**Table S6.** Observed molecular and atomic emission transitions.

| Species | Transition | Wavelength (nm) | Ref. |
| --- | --- | --- | --- |
| CO | d^3^Δ → a^3^Π | 449.2 | (1, 10] |
|  |  | 482.3 |  |
| CO | B^1^∑ → A^1^Π | 517.3 | (1) |
|  |  | 560.6 |  |
|  |  | 605.6 |  |
| H_α_ | 3d^2^D → 2p^2^P^0^ | 656.7 | (2, 3, 10, 14, 15) |
| O | 3s^5^S^0^ → 3p^5^P | 776.5 | (1, 10, 16) |
| O | 3s^3^S^0^ → 3p^3^P | 845.5 | (1, 10, 16) |

**S10 Details of DFT calculations**


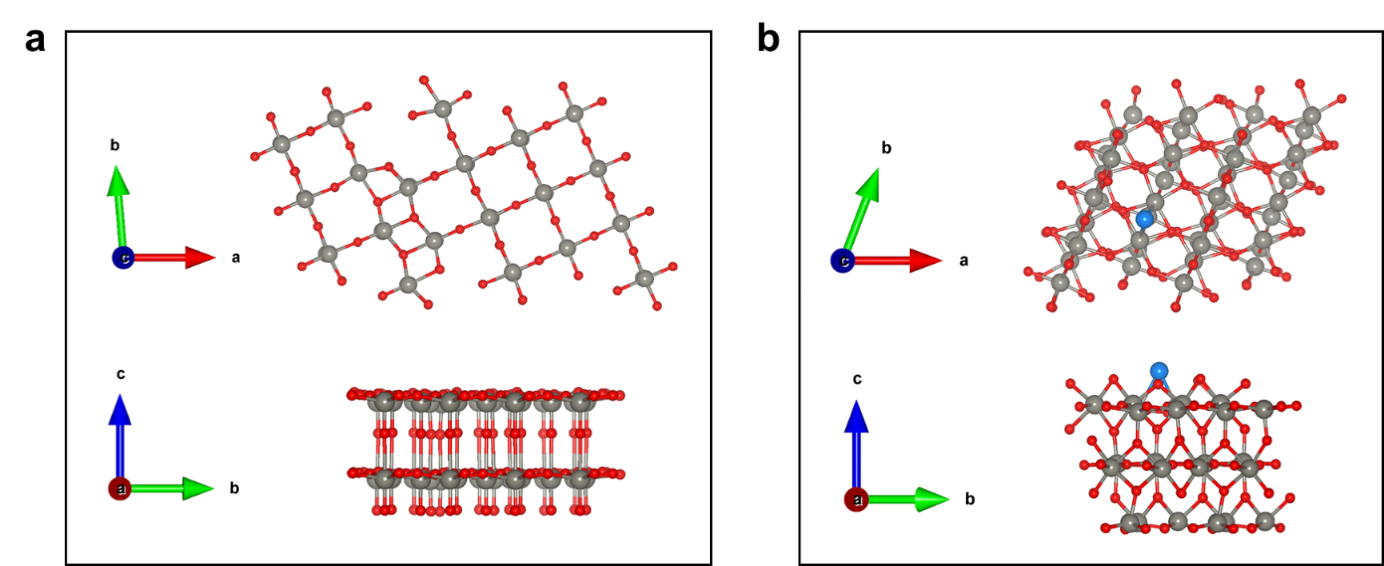


**Figure S36.** Top view (a) and side view (b) of the structures on WO_3-x_ and Pd-WO_3-x_.


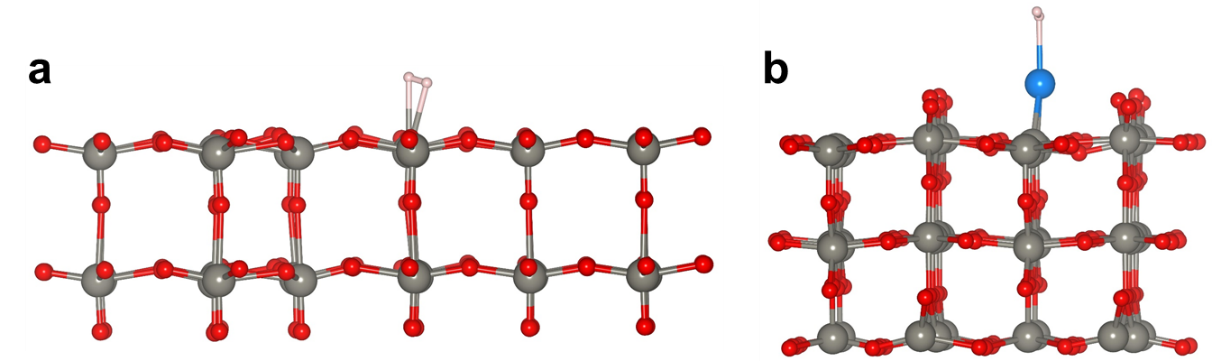


**Figure S37.** Theoretical model of H_2_* on (a) WO_3-x_ and (b) Pd-WO_3-x_. W, Pd, O, and H atoms are shown in grey, blue, red, and white, respectively.


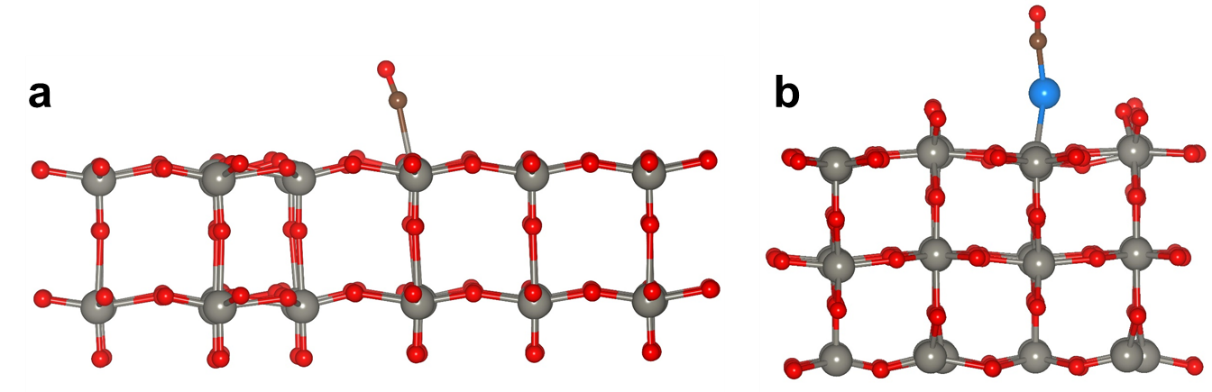


**Figure S38.** Theoretical model of *CO on (a) WO_3-x_ and (b) Pd-WO_3-x_. W, Pd, O, and H atoms are shown in grey, blue, red, and white, respectively.

**Table S7.** Comparison of adsorption energies of CO and H_2_ on WO_3-x_ and Pd-WO_3-x_.

| Catalyst | E_ads_ (CO)  (eV) | E_ads_ (H_2_)  (eV) |
| --- | --- | --- |
| WO_3-x_ | -0.30 | -0.16 |
| Pd-WO_3-x_ | -0.85 | -0.36 |


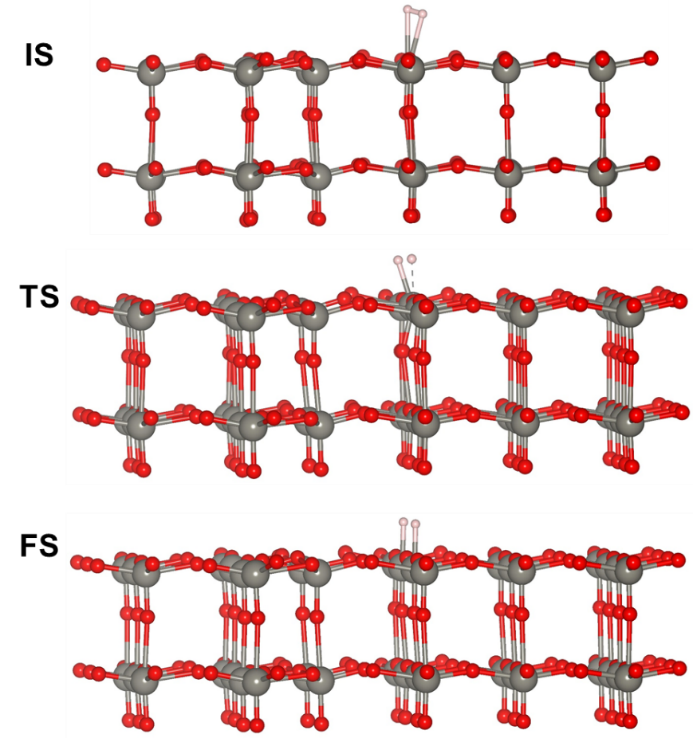


**Figure S39.** Initial state (IS), transition state (TS) and final state (FS) of H_2_ dissociation on WO_3-x_. W, Pd, O, and H atoms are shown in grey, blue, red, and white, respectively.


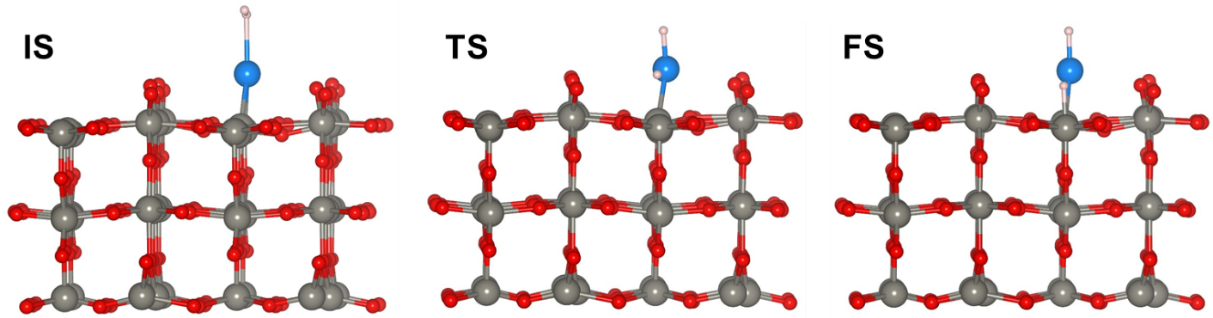


**Figure S40.** Initial state (IS), transition state (TS) and final state (FS) of H_2_ dissociation on Pd-WO_3-x_. W, Pd, O, and H atoms are shown in grey, blue, red, and white, respectively.

**Figure S41.** Projected density of states (PDOS) spectra and corresponding d-band centers of the Pd-WO_3-x_ models.

**References**

(1) Zhu, X.; Liu J. H.; Li, X. S.; Qu, X.; Zhu, A, M. Enhanced effect of plasma on catalytic reduction of CO_2_ to CO with hydrogen over Au/CeO_2_ at low temperature. *J. Energy. Chem*. **2017**, 26 (3), 488-493.

(2) Ashford, B.; Wang, Y. L.; Poh, C. K.; Chen, L. W.; Tu, X. Plasma-catalytic conversion of CO_2_ to CO over binary metal oxide catalysts at low temperatures. *Appl. Catal. B-Environ.* **2020**, 276, 119110.

(3) Yang, Y.; Guo, L.; Luo, S.; Liu, Y.; Song, Y.; Chen, H.; Long, D.; Chen, S.; Wei, Z. Converting CO_2_ to CO via a Plasma-Catalyst Coupled Pathway with Ultrahigh Single-Pass CO_2_ Conversion and∼ 100% CO Selectivity. *ACS Catal.* **2025**, 15 (17), 14955-14965.

(4) Sun, Y. H.; Wu, J. L.; Wang, Y. L.; Li, J. J.; Wang, N.; Harding, J.; Mo, S. P.; Chen, L. M.; Chen, P. R.; Fu, M. L.; Ye, D. Q.; Huang, J.; Tu, X. Plasma-catalytic CO_2_ hydrogenation over a Pd/ZnO catalyst: In situ probing of gas-phase and surface reactions. *JACS Au.* **2022**, 2 (8), 1800–1810.

(5) Bo, Z.; Cao, M. Y.; Wang, Y. L.; Yan, J. H.; Cen, K. F.; Tu, X. Unlocking the Potential of Cu/Ti_3_C_2_T_x_ MXene Catalyst in Plasma Catalytic CO_2_ Hydrogenation. *J. Energy Inst*. **2024**, 115, 101648.

(6) Tian, X.; Wang, D. Y.; Ouyang, B.; Chen, Q.; Liu, Z. W.; Wang, X. W. Plasma-assisted fluidized-bed atomic layer deposition of Pd-Cu nanoparticles on porous powder for CO_2_ hydrogenation. *Plasma Sources Sci. Technol.* **2023**, 32 (4), 045010.

(7) Long, Y. H.; Wang, X. Z.; Zhang, H.; Wang, K. Y.; Ong, W. L.; Bogaerts, A.; Li, K. Z.; Lu, C. Q.; Li, X. D.; Yan, J. H.; Tu, X.; Zhang, H. P. Plasma chemical looping unlocking high-efficiency CO_2_ conversion to clean CO at mild temperatures. *JACS Au.* **2024**, 4 (7), 2462–2473.

(8) Ronda-Lloret, M.; Wang, Y. L.; Oulego, P.; Rothenberg, G.; Tu, X.; Shiju, N. R. CO_2_ hydrogenation at atmospheric pressure and low temperature using plasma-enhanced catalysis over supported cobalt oxide catalysts. *ACS Sustain. Chem. Eng.* **2020**, 8 (47), 17397–17047.

(9) Golubev, O. V.; Maximov, A. L. Dielectric barrier discharge plasma combined with Ce-Ni mesoporous catalysts for CO_2_ splitting to CO. *Plasma Chem. Plasma Process.* **2024**, 44 (6), 2087–2100.

(10) Liu, L.; Zhang, Z. K.; Das, S.; Xi, S. B.; Kawi, S. LaNiO_3_ as a precursor of Ni/La_2_O_3_ for reverse water-gas shift in DBD plasma: Effect of calcination temperature. *Energy Conv. Manag.* **2020**, 206, 112475.

(11) Mei, D. H.; Tu, X. Atmospheric pressure non-thermal plasma activation of CO_2_ in a packed-bed dielectric barrier discharge reactor. *ChemPhysChem.* **2017**, 18 (22), 3253–3259.

(12) Zeng, Y. X.; Chen, G. X.; Liu, B. W.; Zhang, H.; Tu, X. Unraveling temperature-dependent plasma-catalyzed CO_2_ hydrogenation. *Ind. Eng. Chem. Res.* **2023**, 62 (46), 19629–19637.

(13) Liu, L. N.; Das, S.; Chen, T. J.; Dewangan, N.; Ashok, J.; Xi, S. B.; Borgna, A.; Li, Z. W.; Kawi, S. Low temperature catalytic reverse water-gas shift reaction over perovskite catalysts in DBD plasma. *Appl. Catal. B-Environ.* **2020**, 265, 118573.

(14) Chen, G. X.; Georgieva, V.; Godfroid, T.; Snyders, R.; Delplancke-Ogletree, M. P. Plasma assisted catalytic decomposition of CO_2_. *Appl. Catal. B-Environ.* **2016**, 190, 115–124.

(15) Kim, D. Y.; Ham, H.; Chen, X. Z.; Liu, S.; Xu, H. R.; Lu, B.; Furukawa, S.; Kim, H. H.; Takakusagi, S.; Sasaki, K.; Nozaki, T. Cooperative catalysis of vibrationally excited CO_2_ and alloy catalyst breaks the thermodynamic equilibrium limitation. *J. Am. Chem. Soc.* **2022**, 144 (31), 14140–14149.

(16) Lu, X. F.; Yu, L.; Zhang, J. T.; Lou, X. W. Ultrafine dual‐phased carbide nanocrystals confined in porous nitrogen‐doped carbon dodecahedrons for efficient hydrogen evolution reaction. *Adv. Mater.* **2019**, 31 (30), 1900699.
